# Supplementary material for: Frustrated supercritical collapse in tunable charge arrays on graphene
Source: Nat Commun. 2019 Jan 29;10:477. doi: 10.1038/s41467-019-08371-2 (PMC6351629; doi:10.1038/s41467-019-08371-2)
Supplement: Supplementary file 1 — Supplementary Information [file 41467_2019_8371_MOESM1_ESM.pdf]

Supplementary Information

Frustrated supercritical collapse in tunable charge arrays on graphene

Jiong Lu, Hsin-Zon Tsai, Alpin N. Tatan, et al.

Supplementary Note 1 — Experimental materials and methods

Graphene device fabrication. A back-gated graphene/h-BN/SiO_2 device was prepared by overlaying CVD-grown graphene onto hexagonal boron nitride (h-BN) flakes exfoliated onto a SiO2/Si substrate. h-BN flakes were exfoliated onto heavily doped silicon wafers and annealed at 500 °C for several hours in air prior to graphene transfer. The graphene was grown on copper foil by the CVD method and transferred to the h-BN/SiO2 substrate via a polymethyl-methacrylate stamp [1]. Electrical contact was made to the graphene by depositing Ti (10 nm thick)/Au (30 nm thick) electrodes using the stencil mask technique.

Molecular deposition. The graphene device was first annealed in flowing Ar/H_2 gas at 350 °C and subsequently annealed in UHV at $T \approx 350$ °C for several hours until an atomically clean surface was achieved before the deposition of molecules. PCDA and F4-TCNQ were deposited consecutively onto the clean graphene substrate at room temperature using Knudsen cell evaporators in the UHV chamber.

STM/STS measurements. STM/STS measurements were performed under UHV conditions at $T = 5$ K using a commercial Omicron LT STM with tungsten tips. The STM topography was obtained in constant-current mode. The STM tips were calibrated on a Au(111) surface by measuring the Au(111) Shockley surface state before all STS measurements reported here. STS was performed under open feedback conditions by lock-in detection of an alternating tunnel current with a bias modulation of 6–16 mV (r.m.s.) at 400 Hz added to the tunneling bias. The WSxM software was used to process all STM images.

Supplementary Note 2 — STM characterization of the PCDA and F4-TCNQ molecules

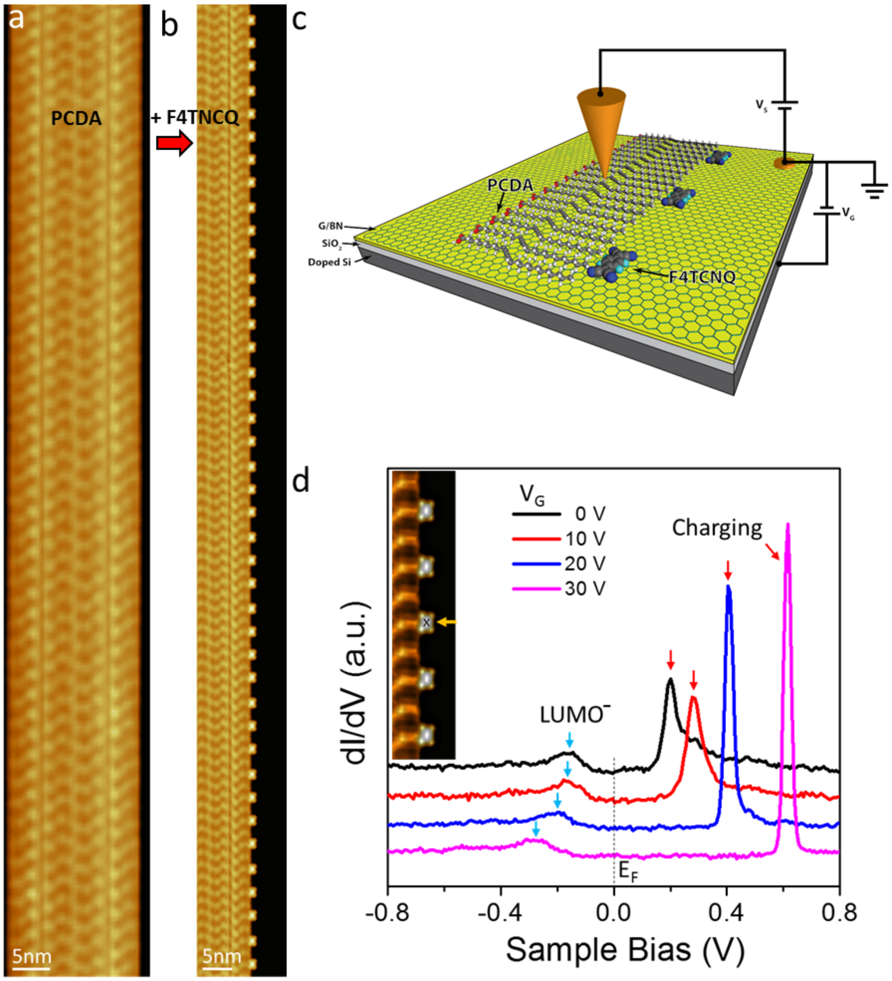

Supplementary Figure 1: (a) Close-up STM image of a PCDA island with straight edges. (b) After deposition of F4-TCNQ, the molecular array is aligned at the edge of these island. c) Schematic illustration of the edge-templated synthesis of F4-TCNQ molecular arrays on a gated graphene FET device. d) dI/dV data acquired directly on a single F4-TCNQ molecule (cross mark in the inset) in the $2a$ molecular array. The tunneling spectra show that individual F4-TCNQ molecules remain negatively charged (the LUMO is below E_F,hence filled) in the range of gate voltages $V_g = 0–30$ V.

Supplementary Note 3 — Distance dependence of the F4-TCNQ LUMO peaks in STS

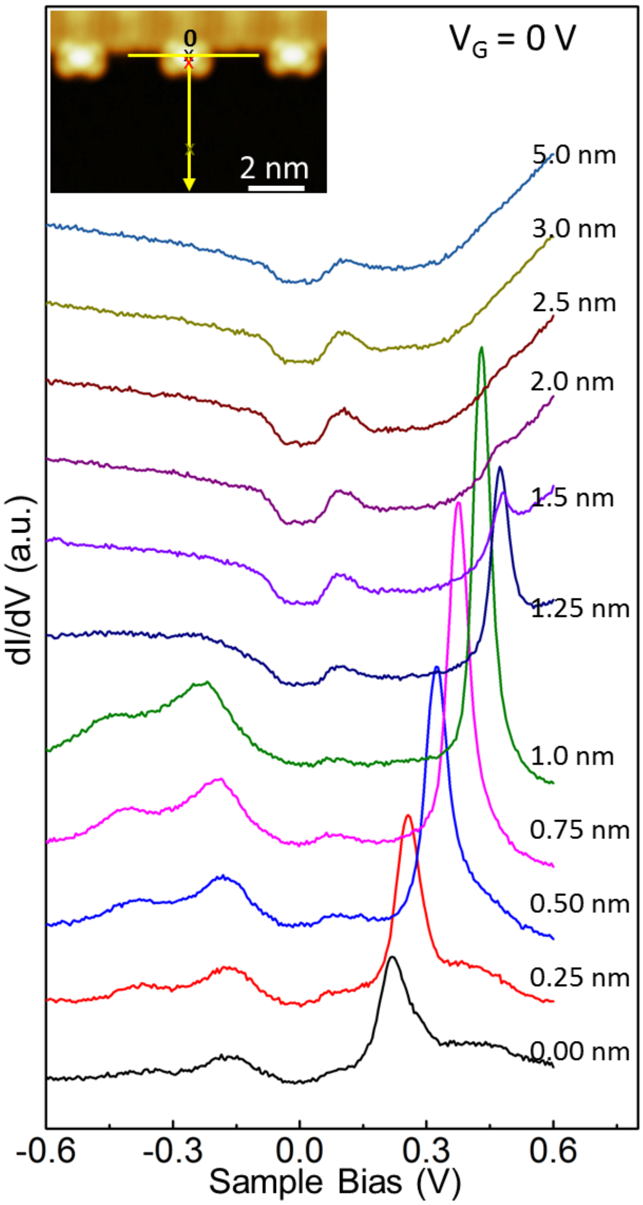

Supplementary Figure 2: Experimental dI/dV taken at $V_g = 0$ V and increasing perpendicular distance from the F4-TCNQ molecule along the direction indicated by the arrow in the inset. The spectral signatures associated with the charging and discharging of the LUMO decay rapidly as soon as the vertical projection of the tip falls outside the molecule at $\sim$1 nm.

Supplementary Note 4 — Distance dependence of the supercritical resonances in STS

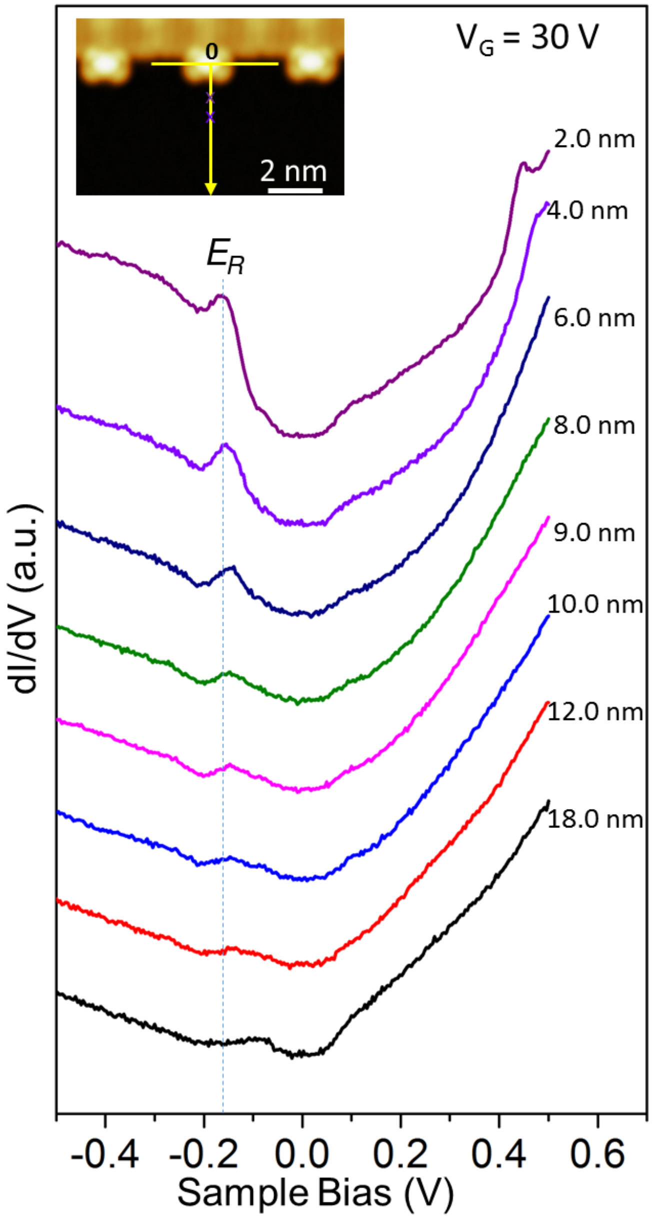

Supplementary Figure 3: Experimental dI/dV taken at $V_g = 30$ V that probes the tunneling into graphene at different perpendicular distances from the F4-TCNQ array along the direction indicated by the arrow in the inset. The vertical dotted line tracks the position of the supercritical resonance at energy E_R. This data set corresponds to the case labeled "$2a$" in Figure 2 of the main text.

Supplementary Note 5 — STS characterization of an isolated F4-TCNQ molecule

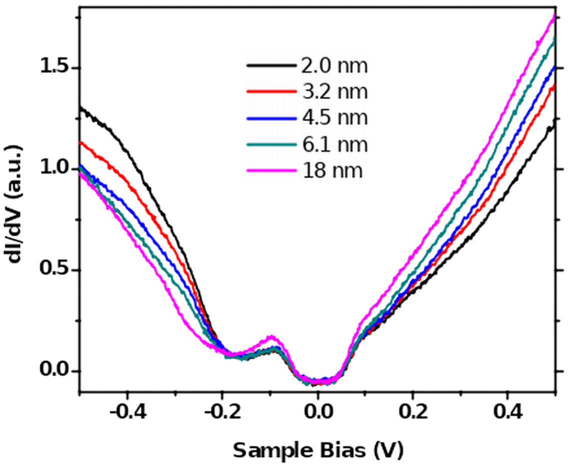

Supplementary Figure 4: Experimental dI/dV measured at different distances from the center of an isolated F4-TCNQ molecule ($V_g = 30$ V). The evolution of the particle-hole asymmetry with distance is consistent with that expected due to the Coulomb field created by a negative point charge at the position of the molecule [7, 10, 8].

Supplementary Note 6 — Characterization of charging rings and associated STS peaks

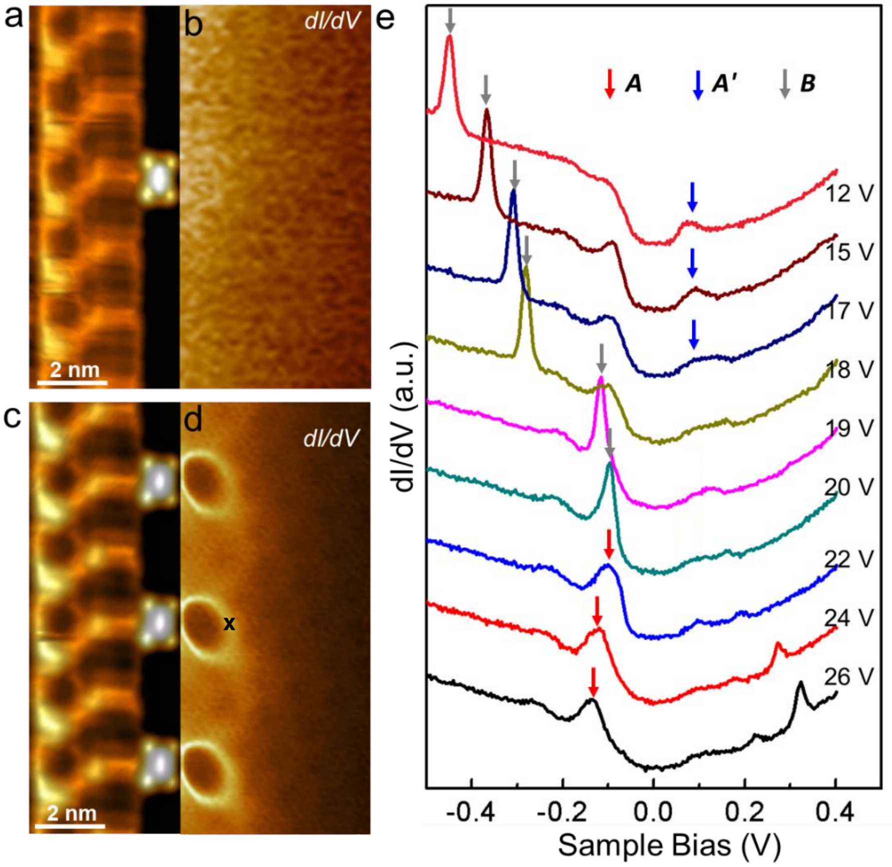

Supplementary Figure 5: Charging and discharging events associated with supercritical states. (a) STM image of a single molecule in a dilute array ($d = 4a$) and (b) the spatial dI/dV map of the adjacent graphene region (gate voltage $V_G = 20$ V and sample bias $V_S = -0.25$ V). (c) STM image of denser ($d = 2a$) array with (d) the corresponding dI/dV map in graphene ($V_G = 20$ V and $V_S = -0.12$ V). Characteristic charging/discharging rings appear reproducibly in the near field of the array. (e) Gate-dependent spectra acquired at a lateral distance of 1.7 nm from the center of a charged F4-TCNQ molecule in the $2a$ array (marked by "x" in panel d).

Details of the charging behavior reported in Fig. 3 of the main text can be best appreciated by analyzing gate-dependent dI/dV point spectra such as those shown in Supplementary Figure 5, acquired with the STM tip held at the edge of the ring marked by "x" in panel d. The combined images in Supplementary Figure 5a-b and Supplementary Figure 5c-d show a continuous region of the surface where the left side is imaged via an STM topograph (showing the molecules adsorbed to graphene underneath and PCDA to the left) and the right side is imaged via a dI/dV map that shows electronic structure in the pristine graphene to the right of the molecular array.

First, note that there is no charging behavior near an isolated molecule, as indicated by the smooth

dI/dV map in Supplementary Figure 5b (recall that the charging state of the molecules does not depend on the inter-molecule separations used in our experiment). This is consistent with the fact that a single molecule behaves as an undercritical Coulomb charge as seen in Supplementary Figure 4 above.

Now, if the resonant peak at energy E_R (Fig. 2 in the main text and Supplementary Figure 6a) reflects quasi-bound supercritical states of graphene, it should track any changes in the position of graphene's Dirac point (E_D) as the latter is varied with the back gate. At the reference gate voltage $V_G = 30\,\mathrm{V}$, graphene is electron-doped and both its Dirac point and resonances lie at negative sample biases (Fig. 2, main text). Decreasing V_G removes electrons and should cause these features to appear at progressively higher sample biases (V_S) in the dI/dV plots. Supplementary Figure 5 indeed shows such progression when $30 > V_G > 10\,\mathrm{V}$ for the group of peaks labeled A. However, at around $V_G = 20\,\mathrm{V}$ there is an evident discontinuity in this evolution: not only does the resonance seem to disappear, but a new, much sharper peak (labeled B in the figure) emerges at high bias (more negative), and with the opposite progression as V_G is reduced further. The disappearance of the resonance is natural in this experiment since at $V_G \simeq 20\,\mathrm{V}$ the resonance (and E_D) merges with the onset of the inelastic gap at $V_S \simeq -65\,\mathrm{meV}$. Logically, these should re-emerge at positive V_S when the the Dirac point is raised above E_F, and they do, as documented by the onset of the broad peaks (labeled A') near $V_S \gtrsim +65\,\mathrm{meV}$ that are clearly visible for all the traces with $V_G < 17\,\mathrm{V}$. The features A and A' are thus simply tracking the position of the resonance at different V_G, and they differ only in that A refers to tunneling from occupied and A' into empty electronic states.

The peaks labeled B, on the other hand, are peculiar and more revealing. Their opposite evolution with V_G signals charging (gain of a localized electron) and discharging (loss of a localized electron) events taking place under the influence of the tip, and are analogous to effects observed in STM measurements near isolated donors or acceptors [2, 3, 4]. The novel aspect here is that graphene, its BN substrate, and the interface are atomically clean, as is evident from our spectroscopic and topographic maps. Consequently, the charging events must be related to the local electronic structure of graphene itself, which is consistent with the existence of the quasi-localized states near the charged array. This is supported by our observation of the hallmark rings in the spatial maps of differential tunneling conductance shown in Supplementary Figure 5 below, which are analogous to those well documented in systems with isolated impurities or adsorbates. These rings are reproducibly centered at positions too far from the F4-TCNQ for them to reflect charging events related to the molecules which remain stably charged in this range of gate voltages (Supplementary Figures 1 and 2). Instead, they provide direct evidence of the charging and discharging of the quasi-localized states whose wave functions have finite probability density in these regions, as per the results of our model calculations.

Typically, systems where this kind of charging ring has been reported always include an impurity or adsorbate on the surface of the electronic system being probed by tunneling spectroscopy

[2, 3, 4] (in these cases, it is the charging of the impurity itself that is responsible for the charging peaks and rings in the tunneling conductance, and they arise from local band bending due to the electrostatic interplay between the tip, the impurity and the surface). The charging events in our system are unique and demonstrate that Coulomb-induced supercritical states in graphene can also be charged and discharged, thereby behaving similarly to localized impurities; except that they are intrinsic to graphene and their wave functions are spread over many lattice sites.

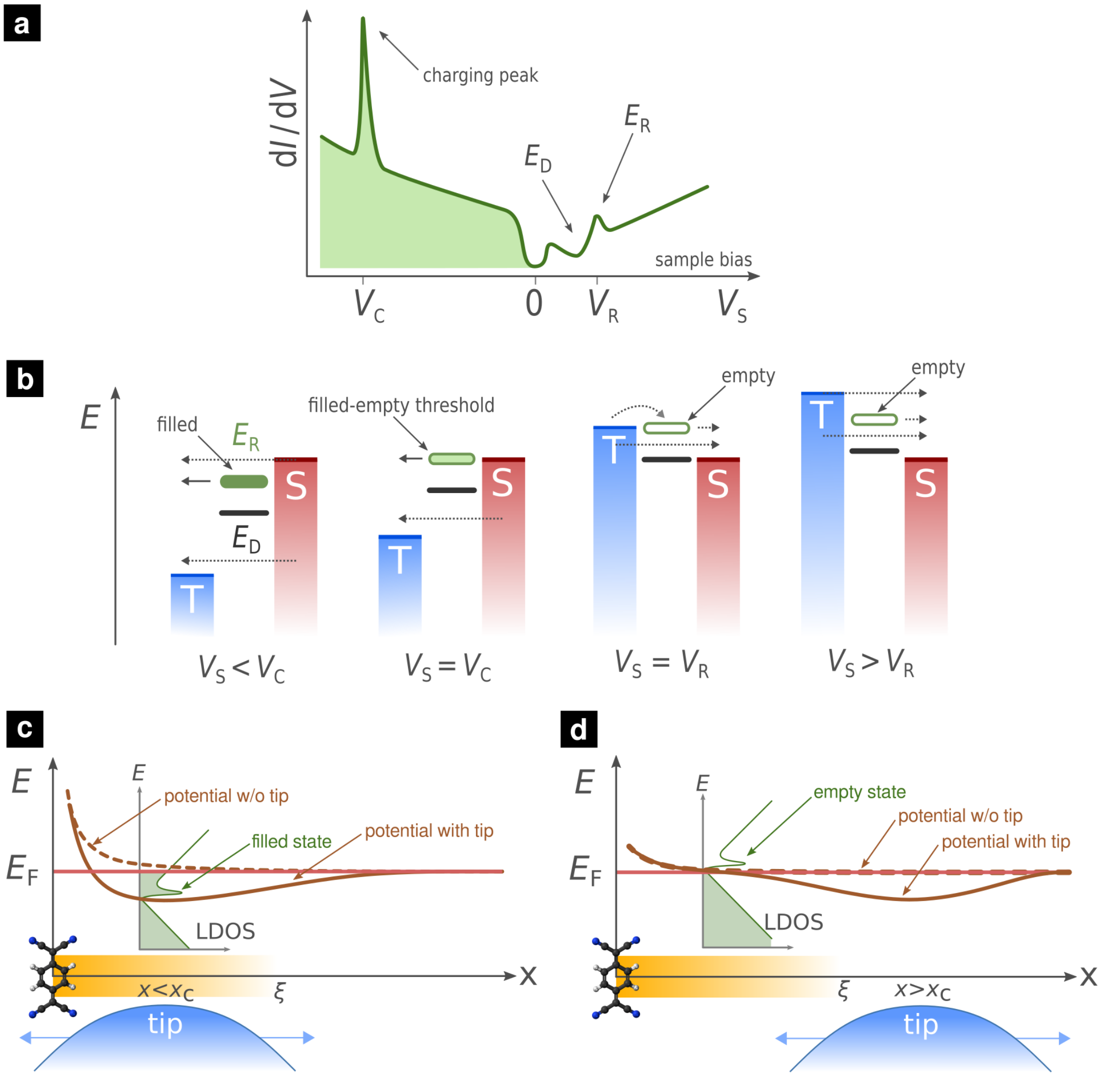

Supplementary Figure 6: Schematic illustration of the charging and discharging events associated with supercritical states (see the text above for a detailed description). (a) Schematic representation of the charging features observed in the experimental dI/dV curves shown in Supplementary Figure 5(e) when the Dirac point (E_D) lies slightly above the Fermi energy (E_F). (b) Illustration of the charging and discharging of the supercritical states when the STM tip is above them. T and S stand for tip and sample, respectively; E_F is the asymptotic Fermi energy in graphene, E_D the graphene Dirac point, and E_R is the energy of the dominant supercritical resonant level in graphene; V_S is the sample bias, V_C the threshold bias at which the level becomes filled/empty, and V_R the bias at which the Fermi energy of the tip and E_R are aligned. Panels (c–d) illustrate the tip-induced band bending when $E_F \approx E_D$ in graphene and the tip is closest to the molecule (c), or beyond the typical decay length (marked ξ in the figures) of the supercritical states. The x axis represents distance to the center of one of the charged molecules, moving perpendicular to the molecular array. The brown-dashed lines sketch the local electrostatic potential in the absence of the tip and the brown-solid ones the total potential in the presence of the tip for $V_S < 0$ at the illustrated positions x.

To understand the process of charging and discharging and the associated emergence of the charging rings, Supplementary Figure 6 schematically illustrates the local band-bending expected to arise due to the tip at different distances from a given F4-TCNQ molecule, as well as different sample biases. Supplementary Figure 6(a) depicts the features in a typical dI/dV trace observed in our measurements [cf. Supplementary Figure 5(e)], highlighting the relative positions of the charging peak (V_C) at negative bias, the Dirac point (E_D), and the dominant supercritical resonance at a positive bias V_R. Band bending also plays a role because these events take place when the Fermi level lies close to E_D and the carrier density is small (nominally zero in ideal graphene).

Supplementary Figure 6(b) shows a simplified energy level diagram for a tip parked at the center of a charging ring at constant V_G. Here, T and S represent the filled states of tip and of the graphene sample far away from the tip, respectively, while E_R and E_D, identify the energies of the resonant state and Dirac point. The supercritical resonance is detected at $V_S \approx V_R$ (third column in panel a) which is positive because the resonant level is empty. It remains empty as the bias crosses over to negative, but its position relative to the asymptotic chemical potential of graphene varies due to the electrostatic potential of the tip. In particular, when $V_S < 0$ the states beneath the tip feel a decrease in potential energy because the tip is at a higher potential than graphene. Consequently, the spectral density is pushed down in energy near the tip apex. If the bias exceeds a negative threshold, V_C, the tip-induced band bending is sufficient to bring the resonance below the chemical potential. The resonant level then becomes populated and contributes a step increase to the tunneling current that translates into a charging peak in the differential conductance. This explains the progression of the experimental traces plotted in Supplementary Figure 5(e) with varying V_G.

The rings observed in Supplementary Figure 5(c,d) arise from the charging behavior described above as the distance between the tip and the localized state is varied. These rings are unusual in that they are centered at locations where there is no external perturbation or impurity in graphene, in contrast to more common observations of charging rings centered at impurity positions [2, 3, 4]. We attribute this to the interplay between the electrostatic potential of each molecule and of the tip, schematically illustrated in Supplementary Figures 6(c,d). The horizontal axis (x) in these panels reflects perpendicular distance to a given F4-TCNQ molecule, with the tip moving perpendicular to the center of a charging ring; V_G is held constant so that the resonance is unoccupied at zero bias. When the tip lies directly above the center of a charging ring the resonant states will be occupied as long as $V_S < V_C$. This is illustrated in the energy-vs-position diagram of Supplementary Figure 6(c), where the solid brown line indicates the total electrostatic potential of the molecule and tip together. When the resonant state is filled, as drawn, the negatively charged molecule is less efficiently screened because of the loss of the supercritical hole (which was positively charged) when the hole state is filled. The charged molecule causes an upward band bending of the graphene electronic structure at the shortest distances to the molecule while the tip induces downward bending in the region directly underneath it. When the tip is

moved closer to the molecule where the bending is dominated by the molecular charge, the resonant level rises above E_F and becomes empty. This discharging event corresponds to the edge of the ring close to the molecule. When the tip is moved away from the molecule, its downward band-bending effect keeps the state filled until the level crosses E_F again at a threshold distance x_C and it becomes empty [Supplementary Figure 6(d)]. This causes the outer edge of the charging ring to appear at x_C. The lateral edges of the charging ring likely arise from the reduction in state density that occurs as the tip moves laterally from the molecule center (cf. Fig. 4a in the main text).

Supplementary Note 7 — Tight-binding model of the charged arrays in graphene

7.1 Hamiltonian and related parametrization

We consider non-interacting electrons in graphene described by the following p_z-derived single orbital tight-binding Hamiltonian [5, 6]

$$H = -t \sum_{\langle i,j \rangle} c_i^\dagger c_j + \text{H.c.} + \sum_i V(\boldsymbol{r}_i)\, c_i^\dagger c_i, \tag{1}$$

where $t = 2.7$ eV represents the nearest neighbor hopping amplitude, $c_i^\dagger$ is the second quantized creation operator for an electron at site i of the underlying honeycomb lattice, and

$$V(\boldsymbol{r}) \equiv \sum_{n=1}^{N} v(\boldsymbol{r} - \boldsymbol{R}_n) \tag{2}$$

is the total potential energy at point $\boldsymbol{r}$ arising from the distribution of N charged F4-TCNQ molecules located at positions $\boldsymbol{R}_n$.

To account for screening by the finite electronic density in our gated devices while, at the same time, permitting expedient numerical calculations in our very large lattices, the Coulomb potential created by each molecule is modeled as (details in section below)

$$v(\boldsymbol{r}) \equiv \frac{Ze^2}{\kappa_g \kappa_e r} \left[\frac{1}{1 + (r/\lambda_s)^2} \right], \tag{3}$$

where κ_e and κ_g are the dielectric constants contributed by the environment (BN substrate and air) and graphene itself. The screening length λ_s is taken as a fitting parameter in our model.

For the numerical implementation, it is convenient to define a dimensionless version of H, which

we write as

$$\tilde{H} \equiv t^{-1} H = -\sum_{\langle i,j \rangle} c_i^\dagger c_j + \text{H.c} + \sum_i \tilde{V}(\boldsymbol{r}_i)\, c_i^\dagger c_i\,, \tag{4}$$

with

$$\tilde{V}(\boldsymbol{r}) \equiv \sum_{n=1}^{N} \tilde{v}(\boldsymbol{r} - \boldsymbol{R}_n), \qquad \tilde{v}(\boldsymbol{r}) \equiv \frac{Q}{r}\left[\frac{1}{1+(r/\lambda_s)^2}\right], \tag{5}$$

and lengths are now expressed in units of the carbon-carbon distance in graphene, $c \simeq 0.142$ nm. The adimensional potential strength is given explicitly by

$$Q \equiv \frac{Ze^2}{\kappa_g \kappa_e t c} = \frac{3Ze^2}{2\kappa_g \kappa_e \hbar v_F} = \frac{3}{2} Z \frac{\alpha_0}{\kappa_e \kappa_g} = \frac{3}{2} Z\alpha. \tag{6}$$

In this sequence of definitions, $v_F \equiv 3tc/2\hbar$ is the Fermi velocity, $\alpha_0 \equiv e^2/\hbar v_F \simeq 2.5$ is the bare (i.e., in vacuum) fine structure constant in graphene, and α the actual fine structure constant corrected by the dielectric properties of the environment and graphene itself. A contour plot of the potential (5) is given in Supplementary Figure 7.

In this tight-binding formulation, we use the parameter Q instead of $Z\alpha$ itself to quantify the strength of the coupling between electrons in graphene and the external Coulomb field. A *single*, *unscreened* Coulomb charge is supercritical if [7, 8, 11]

$$Z\alpha > 0.5 \qquad \text{(supercritical regime)} \tag{7}$$

which, in terms of the parameter Q, translates to

$$Q > Q_c \equiv 0.75 \qquad \text{(supercritical regime).} \tag{8}$$

Note that the supercritical threshold of such an isolated Coulomb charge is determined by the product of Z and α ($Z\alpha = 0.5$), and not by either independently. Once the dielectric environment is specified, one can compute the effective fine-structure constant defined in eq. (6) as $\alpha \equiv \alpha_0/(\kappa_e \kappa_g)$ and obtain the critical Z for those dielectric conditions as

$$Z_c = \frac{1}{2\alpha} \tag{9}$$

The relevant estimate for the case of the F4-TCNQ experiment discussed in the main text is discussed in detail in Supplementary Note 11.

7.2 Placement of the charge centers

When charged, each F4-TCNQ molecule is considered to act as a point Coulomb center of effective valence Z_{eff} and contributing the electrostatic potential given by equation (3). We verified that

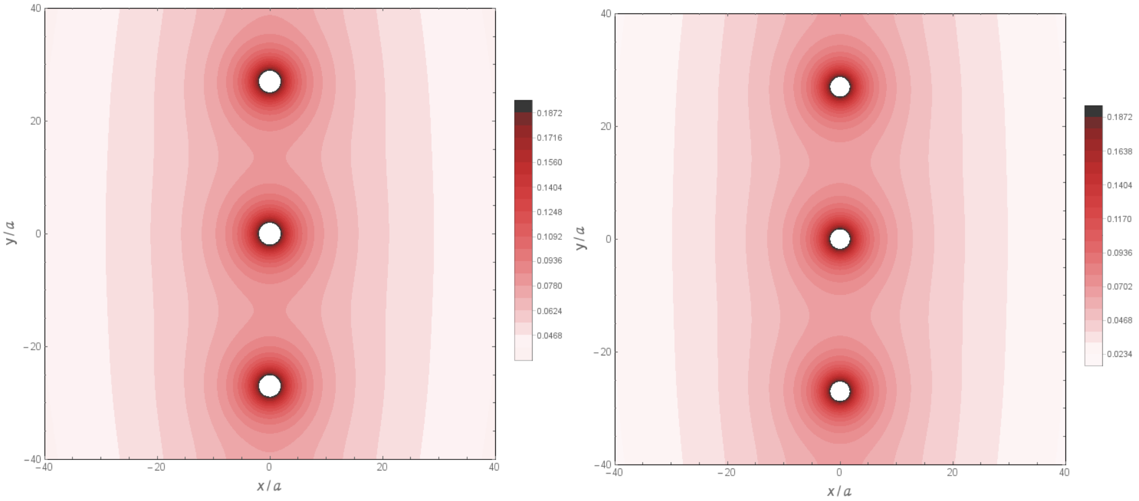

Supplementary Figure 7: Contour plot of the electrostatic potential $\tilde{V}(\boldsymbol{r})$ in eq. (5) for charges separated by $3.85\,\mathrm{nm}$, $Q = 0.5$, and screening parameters $\lambda_s = \infty$ (left) and $\lambda_s = 10\,\mathrm{nm}$ (right). Coordinates are given in units of the carbon-carbon distance in graphene, $c \simeq 0.142$ nm (hence, $10\,\mathrm{nm} \simeq 70c$).

there is no difference in the results between assuming point charges at the geometrical centers of each molecule or a uniform distribution extended over the area of each molecule; not surprising given that the distances where the LDOS is probed are considerably larger than the size of the molecules, whence only the monopole contribution from each molecular charge is relevant. Charges are placed at the center of an hexagon in the honeycomb lattice, arranged along a line parallel to the armchair direction, similarly to the relative orientation of the array and graphene's crystal directions in the experiments. The distance between neighboring charges is chosen to give the closest match to the experimental periods, and the array itself is placed in the center of our simulation cell. This layout is schematically illustrated in Supplementary Figure **8**.

Our LDOS calculations were performed on a lattice with 1000×1000 sites ($\simeq 123 \times 213\,\mathrm{nm}^2$), and an array of charges that spans total length $L \simeq 85$ nm. These dimensions are large enough to: (i) have an effective translation invariance in the vicinity of the central charges, (ii) to have a large enough central region that is insensitive to the finite size of the charged array because its length is much larger than the typical screening distances, (iii) to allow one to reach the asymptotic distances from the array, where the mildly screened potential has no effect, and also (iv) to have an overall field of view that safely encompasses the actual magnitude of the areas mapped experimentally.

For the purposes of the comparison with the experimental dI/dV traces in Fig. 2 of the main text, the local spectrum is computed at lattice sites x that run perpendicularly to the charge array.

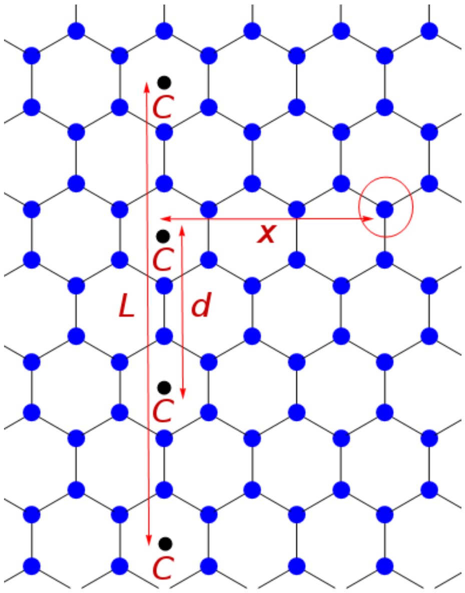

Supplementary Figure 8: Illustration of the placement of the point charges that represent each F4-TCNQ molecule with respect to the graphene lattice where any pair of carbon atoms is separated by $c \simeq 0.142$ nm. The black dots labeled "C" mark the positions of the point charges that always reside at the center of one hexagon. The labels mark the distance between charges (d, in our model is always multiple of $3c$), the total length of the array (L), as well as the distance (x) measured perpendicularly to the array where the LDOS is computed.

7.3 Calculation of the local density of states

The bare LDOS, $\rho(E, \boldsymbol{r})$ at position $\boldsymbol{r}$ and energy E can be expressed in terms of the one-particle Green's function as

$$\rho(E, \boldsymbol{r}) = -\frac{1}{\pi} \lim_{\eta \to 0^+} \operatorname{Im} G_{\boldsymbol{r}\boldsymbol{r}}(E + i\eta), \tag{10}$$

where the local Green's function is formally defined as the expectation value of the resolvent operator for the Schrödinger equation:

$$G_{\boldsymbol{r}\boldsymbol{r}}(E) \equiv \langle \boldsymbol{r} | \frac{1}{E - \hat{H}} | \boldsymbol{r} \rangle. \tag{11}$$

In lattice model such as (4) where the Hamiltonian is represented by a matrix H_{ij} in the tight-binding basis, the lattice Green's function is defined in corresponding way by

$$G_{ii}(E) \equiv \left[\frac{1}{E - H} \right]_{ii}, \tag{12}$$

and thus the LDOS at lattice site i is given by

$$\rho_i(E) = -\frac{1}{\pi} \operatorname{Im} G_{ii}(E + i\eta). \tag{13}$$

Since the matrix inversion implied by equation (12) is a very costly numerical operation and since we, in addition, need to use very large system sizes, we employ Haydock's scheme to compute $G_{ii}(E)$ directly in recursive scheme that bypasses the inversion of the Hamiltonian matrix. The method involves two numerically very efficient steps [12]. A conventional Lanczos iteration is performed first to extract tridiagonal representation of H to a desired level of truncation:

$$H \mapsto \begin{bmatrix} a_0 & b_1 & 0 & \cdots & 0 \\ b_1 & a_1 & b_2 & \cdots & 0 \\ 0 & b_2 & a_2 & \cdots & \vdots \\ \vdots & \vdots & \vdots & \ddots & b_{N-1} \\ 0 & 0 & \cdots & b_{N-1} & a_{N-1} \end{bmatrix}. \tag{14}$$

If the starting vector for the tridiagonalization is chosen as that representing the electron at lattice site i, the tridiagonal coefficients derived from it define a recursion relation for successively more accurate approximations to the local Green's function at site i:

$$G_{ii} \simeq G_{ii}^{(0)}(E), \qquad \text{where} \qquad G_{ii}^{(n)}(E) = \frac{1}{E - a_n - b_{n+1}^2 G_{ii}^{(n+1)}(E)}. \tag{15}$$

Of the various methods to handle the termination of this continued fraction [13], we chose to simply set

$$G_{ii}^{(N-1)}(E) = \frac{1}{E - a_{N-1}}, \tag{16}$$

and use an explicitly finite broadening parameter, $\eta = 0.005\,t \simeq 13.5\,\text{meV}$. Our calculations have been done with a tridiagonalization truncated at $N = 2000$, which is sufficient to obtain the resolution dictated by our choice of η.

In addition to its numerical expediency, the critical advantage of this method it that it does not require storing the matrix representation of the Hamiltonian. This permits one to calculate the LDOS in systems of arbitrarily large size, since the storage limits scale linearly rather than quadratically in the number of lattice sites.

7.4 Exact diagonalization calculations

In these calculations, we build the complete matrix representing the Hamiltonian in equation (4) and determine the complete set of its eigenvalues and eigenvectors with resort to the LAPACK suite of numerical routines [14].

As we want exact spectra and wavefunctions, there are stringent storage constraints imposed by the need to store the full matrix and we used systems of up to 200×160 sites. Since we are

foremost interested in the spatial distribution of the quasi-localized states which decay relatively fast away from the array of charges, we determined that lattices of dimension 160×100 ($\simeq 19 \times 21\,\mathrm{nm}^2$) are large enough to both comfortably avoid finite-size artifacts and accommodate the typical screening lengths used in relation to the experiments.

Supplementary Note 8 — Screened Coulomb potential

As we are interested in how the electron gas in graphene screens the external Coulomb field generated by each charged F4-TCNQ molecule, we consider the static dielectric constant which, in the random phase approximation (RPA), is given by

$$\epsilon(\boldsymbol{q}) = 1 + v(\boldsymbol{q})\,\Pi(\boldsymbol{q}). \tag{17}$$

In this expression $\Pi(\boldsymbol{q})$ represents the (Lindhard) electronic polarizability at zero frequency, and is given explicitly in the Dirac approximation for graphene by [11, 17, 18, 19] (at $T = 0$)

$$\Pi(\boldsymbol{q}) = \rho(E_F) - \rho(E_F)\,\theta(q - 2k_F)\left[\frac{1}{2}\sqrt{1 - \left(\frac{2k_F}{q}\right)^2} + \frac{q}{4k_F}\sin^{-1}\!\left(\frac{2k_F}{q}\right) - \frac{\pi q}{8k_F}\right], \tag{18}$$

where $\rho(E_F) \equiv \frac{g_s g_v k_F}{2\pi\hbar v_F}$ is the DOS at the Fermi level, $g_s = g_v = 2$ is the spin and valley degeneracy, and $E_F = v_F \hbar k_F$ the Fermi energy.

If the bare Coulomb field of a unit charge is written in real and Fourier space as

$$v^{(0)}(\boldsymbol{r}) = \frac{e^2}{\kappa_s r} \qquad \longleftrightarrow \qquad v^{(0)}(\boldsymbol{q}) = \frac{2\pi e^2}{\kappa_s q}, \tag{19}$$

where κ_s is an overall environmental dielectric constant, the screened potential is given by

$$v(\boldsymbol{q}) = \frac{v^{(0)}(\boldsymbol{q})}{\epsilon(\boldsymbol{q})}. \tag{20}$$

As our calculations are done in real space, directly in the honeycomb lattice, we need the real space representation of this screened potential, which corresponds to

$$v(\boldsymbol{r}) = \frac{1}{4\pi^2}\int d\boldsymbol{q}\,\frac{v(\boldsymbol{q})\,e^{i\boldsymbol{q}\cdot\boldsymbol{r}}}{1 + v(\boldsymbol{q})\,\Pi(\boldsymbol{q})}. \tag{21}$$

In the Thomas-Fermi limit valid far from the charge center,

$$v_{\mathrm{TF}}(\boldsymbol{q}) \equiv \lim_{q\to 0}\frac{v^{(0)}(\boldsymbol{q})}{\epsilon(\boldsymbol{q})} = \frac{2\pi e^2}{\kappa_s(q + q_{\mathrm{TF}})}, \qquad q_{\mathrm{TF}} \equiv \frac{4e^2 k_F}{\kappa_s \hbar v_F} = 4\alpha k_F, \tag{22}$$

which becomes, in real space,

$$v_{\mathrm{TF}}(\boldsymbol{r}) = \frac{e^2}{\kappa_s r} f(q_{\mathrm{TF}} r), \qquad f(x) \equiv 1 - \frac{\pi x}{2}\Big[\boldsymbol{H}_0(x) - \mathbf{Y}_0(x)\Big]. \tag{23}$$

The asymptotic behavior of the Struve and Bessel functions appearing in the screening function ($f(x) \sim 1/x^2$) dictates the decay of the potential at long distances [20]:

$$v_{\mathrm{TF}}(\boldsymbol{r}) \sim \frac{e^2}{\kappa_s r} \frac{1}{(q_{\mathrm{TF}} r)^2}, \qquad r \gg q_{\mathrm{TF}}^{-1}. \tag{24}$$

In the opposite limit of short distances, the integral in (21) approximates to

$$v(\boldsymbol{r}) \approx \frac{e^2}{\kappa_s r}\left(1 + \frac{\pi e^2}{2\kappa_s \hbar v_F}\right)^{-1} = \frac{e^2}{\kappa_s \kappa_g r}, \qquad r \ll q_{\mathrm{TF}}^{-1}. \tag{25}$$

The factor $\kappa_g \equiv 1 + \frac{\pi e^2}{2\kappa_s \hbar v_F} = 1 + \frac{\pi\alpha}{2}$ is a constant and reflects the fact that, although in a trivial way, inter-band transitions in graphene always contribute to reduce the Coulomb interaction by this factor, even in the limit of zero doping [11]. That the potential preserves its $1/r$ form at short distances is not surprising on physical grounds. The presence of graphene merely introduces and additional "short-distance" dielectric constant κ_g [19].

The two asymptotic regimes described by (24) and (25) can be interpolated with a single screening function,

$$v(\boldsymbol{r}) \simeq \frac{e^2}{\kappa_s \kappa_g r} F(q_{\mathrm{TF}} r), \qquad F(x) \equiv \frac{1}{1 + x^2/\kappa_g}. \tag{26}$$

This is the form of the potential stated earlier in equation (3).

This interpolation not only ensures the correct asymptotic behavior in the two limits, but constitutes an excellent numerical approximation to the exact result given by (21), as illustrated in the comparison shown in Supplementary Figure **9**. Thus, for expediency in our numerical calculations, we have assigned to each charged F4-TCNQ a screened potential described by equation (26).

The screening function can be expressed in terms of a characteristic screening length λ_s:

$$F(q_{\mathrm{TF}} r) = \frac{1}{1 + r^2/\lambda_s^2}, \tag{27}$$

where, in principle, λ_s should be strictly given by

$$\lambda_s \equiv \frac{1}{q_{\mathrm{TF}}\sqrt{\kappa_g}} = 4\alpha\sqrt{\pi n_e}, \tag{28}$$

which is an explicit function of the carrier density in graphene (n_e). Now, on the one hand, in our experiments there is evidence of band bending effects as discussed in the main text (of which

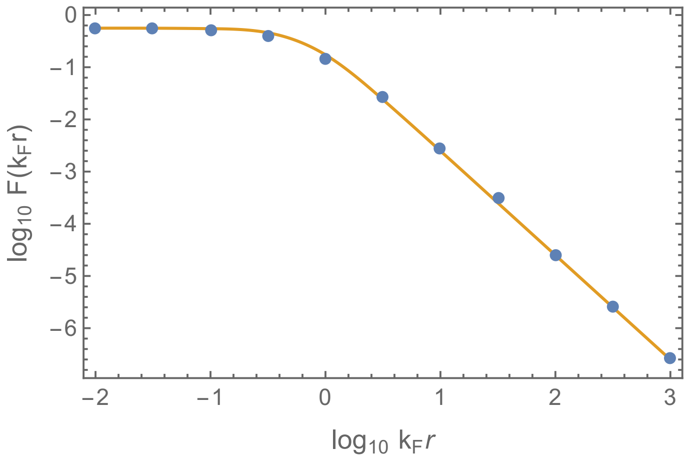

Supplementary Figure 9: Comparison of the simplified screening function in real space defined in equation (26) (line) with the exact screening function arising from the Fourier integral (21) in the RPA (dots).

the charging/discharging rings are a direct consequence). Combined with the proximity of the resonant features to the inelastic gap in dI/dV and the spectrum reconstruction that takes place near the Dirac point (E_D) due to the supercritical resonances, this precludes an unambiguous determination of E_F. On the other hand, the self-consistent screening induced by these quasi-localized supercritical states is expected to modify the effective decay length in a non-trivial way compared with the perturbative RPA calculation that yields (26) [8, 11, 21]. Therefore, rather than fixing λ_s in terms of the carrier density of graphene as per (28) (which is difficult to determine with certainty under the current measurement conditions), we treat it as a fitting parameter as discussed in the main text.

We note however that the essential physics of the supercritical regime (namely the stabilization of the quasi-bound states within a short distance from the charges) is dictated by the short range behavior of the potential which, according to (26) is insensitive to the screening length.

Supplementary Note 9 — Simulated dI/dV curves from the bare LDOS calculations

It is well documented that the protocol used in our experiments to calibrate the scanning tunneling microscope (STM) tips results in reproducible high sensitivity of the measurements to inelastic tunneling assisted by $\boldsymbol{K}$ point phonon modes of graphene [15]. This new tunneling channel opens at biases of magnitude 63 ± 2 meV, at which point the differential tunneling conductance undergoes an abrupt and large step increase, leaving an apparent gap (that we designate as "inelastic gap") in the dI/dV traces in the window of bias voltages $-63 < V_s < +63$ V.

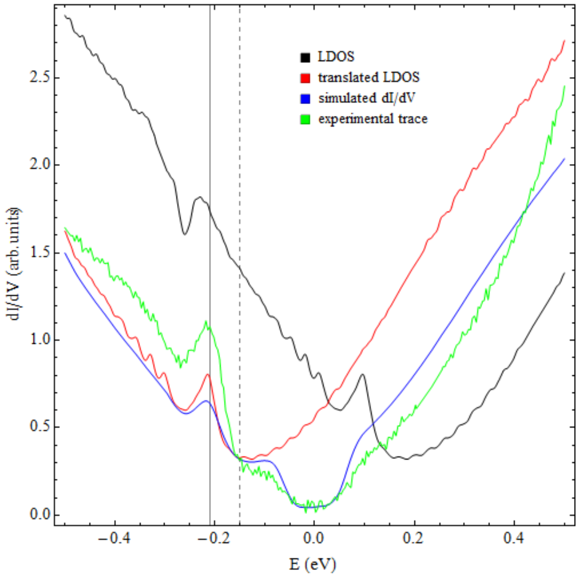

Supplementary Figure 10: Illustration of the procedure to adjust the theoretical dI/dV curves to the experimental traces. In this example, we choose the case where dI/dV is probed at distance of 3.2 nm from an array of charges with period 3.85 nm (the experimental case "$2a$") and total length 85.2 nm. The potential energy is the one described by equations (2) and (3), with $Q = 0.5$ and $\lambda_s = 10\,\text{nm}$. The green curve is the experimental trace, black the bare LDOS, red the LDOS shifted to account for finite doping combined with the band-bending correction, and blue the simulated dI/dV based on the red theoretical trace.

To accommodate this effect for a direct quantitative comparison of the dI/dV traces with our calculated LDOS, we have followed the procedure outlined in reference [16] to model the increase in the tunneling current. The total dI/dV signal is given by

$$\frac{dI}{dV}(V,\boldsymbol{r}) = \frac{dI}{dV}(V,\boldsymbol{r})\bigg|_{\text{elastic}} + \frac{dI}{dV}(V,\boldsymbol{r})\bigg|_{\text{inelastic}}, \tag{29}$$

where the elastic contribution is simply proportional to the local DOS, $\rho(E, \boldsymbol{r})$,

$$\frac{dI}{dV}(V,\boldsymbol{r})\bigg|_{\text{elastic}} \propto \rho(V,\boldsymbol{r}), \tag{30}$$

and

$$\frac{dI}{dV}(V,\boldsymbol{r})\bigg|_{\text{inelastic}} = \sum_{i=1}^{2} \frac{\lambda_i}{\sigma_i\sqrt{2\pi}} \left| \int_0^{eV} d\varepsilon\,(eV-\varepsilon)\, e^{-(|\varepsilon|-\hbar\omega_i)^2/2\sigma_i^2}\, \frac{dI}{dV}(V,\boldsymbol{r})\bigg|_{\text{elastic}} \right|. \tag{31}$$

The parameters characterizing the two dominant inelastic excitations are their energy ($\hbar\omega_1 =$ 63 meV, $\hbar\omega_2 = 150$ meV), their width ($\sigma_1 = \sigma_2 = 17$ meV) and their strength ($\lambda_1 = 10$, $\lambda_2 = 0.67$) [16]. In our calculations, the simulated $\frac{dI}{dV}(V,\boldsymbol{r})$ curves are obtained by replacing the elastic term with the bare LDOS calculated at the tight-binding level. The inelastic term over-

whelmingly dominates the total current above the inelastic gap, and the presence of the Gaussian in the convolution-type integral of equation (31) smoothens the finer spectral features of the bare LDOS considerably (Supplementary Figure **10**).

An additional step is necessary before we directly compare the simulated and experimental curves. Since our calculation of the LDOS does not include electron-electron interaction effects (other than using a screened Coulomb law for the potential created by the charged molecules), the LDOS curves show a strong band-bending as a function of distance to the array of charges because there is no self-consistency to correct the local potential energy as would be the case in the real system. This artificially excessive band-bending is corrected by translating the simulated dI/dV curves in energy so that the position of the dip near the onset of the inelastic gap coincides with the one in the corresponding measurement. To be specific, we show in Supplementary Figure **10** an actual example of this procedure:

1. The black curve is the bare, single-particle LDOS calculated at the probing site for this charge distribution; its minimum is lies around 0.15 eV due to the strong local band bending effect near the charges on the F4-TCNQ molecules (that are repulsive from the point of view of the electrons).

2. In order to simultaneously compensate for the absence of self-consistent screening in this calculation and describe the finite E_F in the experimental system, the bare LDOS (black) is translated leftwards until its dip coincides with the bias voltage of the experimental dip. The result is the red curve in Supplementary Figure **10**. The vertical dashed line helps identifying the points where the raise in the theoretical LDOS matches the corresponding feature in the experimental dI/dV.

3. The red trace is then used in eqs. (31) and (29) to simulate the effect of the inelastic channel and the "gap" feature in the dI/dV spectrum around $V_b = 0$. The result is the blue curve. The solid vertical line helps verifying that the peak position in the simulated dI/dV coincides with the experiment.

This procedure is done only once for each array of charges by selecting the dI/dV trace taken closest to the array. The offset determined in step 2 for the closest trace is then applied to all the traces taken at distances farther away, without further adjustment (i.e., the same offset is applied to all simulated dI/dV curves).

It is noticeable in the figure that the bare LDOS traces (red) have a richer structure and modulation at energies to the left of the dominant resonance, in comparison with the smoother behavior of the final simulated dI/dV (blue). This is due to the fact that the inelastic contribution computed according to (31), in effect, broadens the bare LDOS by an amount determined by the parameters σ_i due to the presence of the Gaussian function under the integral.

Supplementary Note 10 — Decay of the computed LDOS and supercritical wavefunctions

In order to complement the data shown in Fig. 2 of the main text for the simulated dI/dV traces as a function of distance, Supplementary Figure 11 shows how the maximum at the resonant peak in the LDOS decays with perpendicular distance to the charged array (along x in Supplementary Figure 8) for the case of the array with $d = 2a$, which exhibits the strongest resonance in the experiments.

Generically, one can express the LDOS $N(E, \boldsymbol{r})$ in terms of individual normalized eigenstate wave functions as

$$N(E, \boldsymbol{r}) = \sum_{E_n} |\psi_{E_n}(\boldsymbol{r})|^2 \, \delta(E - E_n), \tag{32}$$

where N_c is the total number of carbon atoms. Under the approximation that a local resonance with energy E_r is primarily contributed by one state, we can write

$$N(E, \boldsymbol{r}) \simeq \sum_{E_n \neq E_R} |\psi_{E_n}(\boldsymbol{r})|^2 \, \delta(E - E_n) + |\psi_{E_r}(\boldsymbol{r})|^2 \simeq N^{(0)}(E, \boldsymbol{r}) + |\psi_{E_r}(\boldsymbol{r})|^2. \tag{33}$$

Therefore, the change in LDOS, $\Delta N(E, \boldsymbol{r})$, induced by the perturbation that caused the resonant state provides a measure of the probability distribution of the associated wave function:

$$\Delta N(E, \boldsymbol{r}) \equiv N(E, \boldsymbol{r}) - N^{(0)}(E, \boldsymbol{r}) \simeq |\psi_{E_r}(\boldsymbol{r})|^2. \tag{34}$$

To illustrate this, the last panel of Supplementary Figure 11 shows a comparison between the distance dependence of $\Delta N(E, \boldsymbol{r})$ (computed using the recursive method) and the decay of $|\psi_{E_r}(\boldsymbol{r})|^2$ for the specific wave function shown in Supplementary Figure 12 below (computed by exact diagonalization of the tight-binding Hamiltonian). The decay is very approximately the same, as anticipated from the above, which also provides a consistency check to our different numerical calculations.

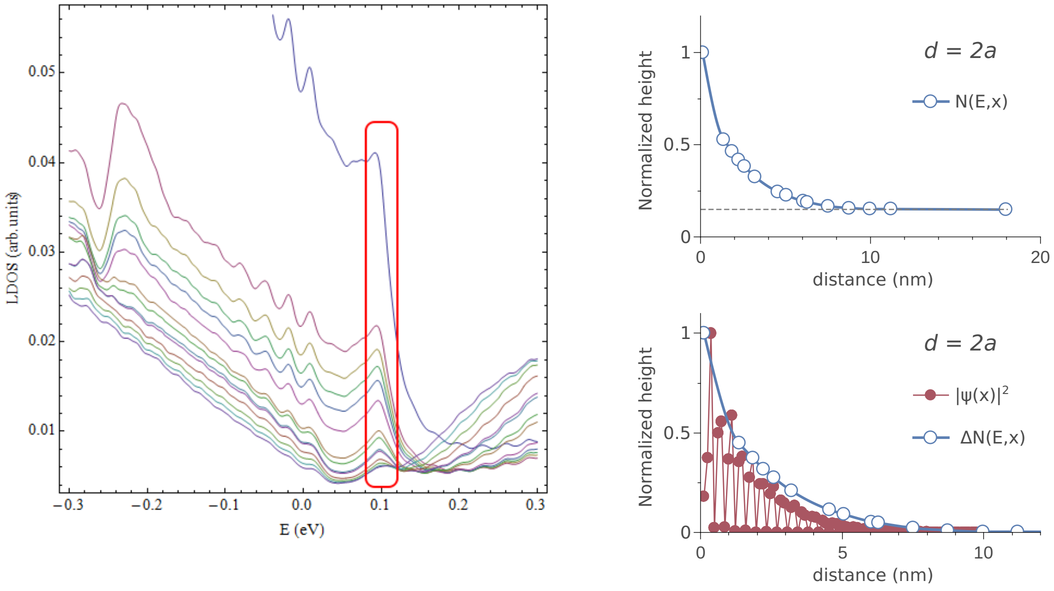

Supplementary Figure 11: Left: the bare LDOS curves at different perpendicular distances to the array of charges (along a line intersecting one of them). The rectangle highlights the position of the resonant peak whose magnitude is plotted in the right-hand panel.

Right: the top plot shows the magnitude of the LDOS at the resonant peak as a function of perpendicular distance to the array. The dashed horizontal line is drawn to identify the asymptotic value. The bottom plot shows the same data (subtracted from the asymptotic value) side-by-side with the probability distribution of the eigenstate shown in Fig. 4(a) of the main text. The calculations were done for the same parameters as in Supplementary Figure 10 (array with $d = 2a$, $Q = 0.5$ and $\lambda_s = 10\,\mathrm{nm}$).

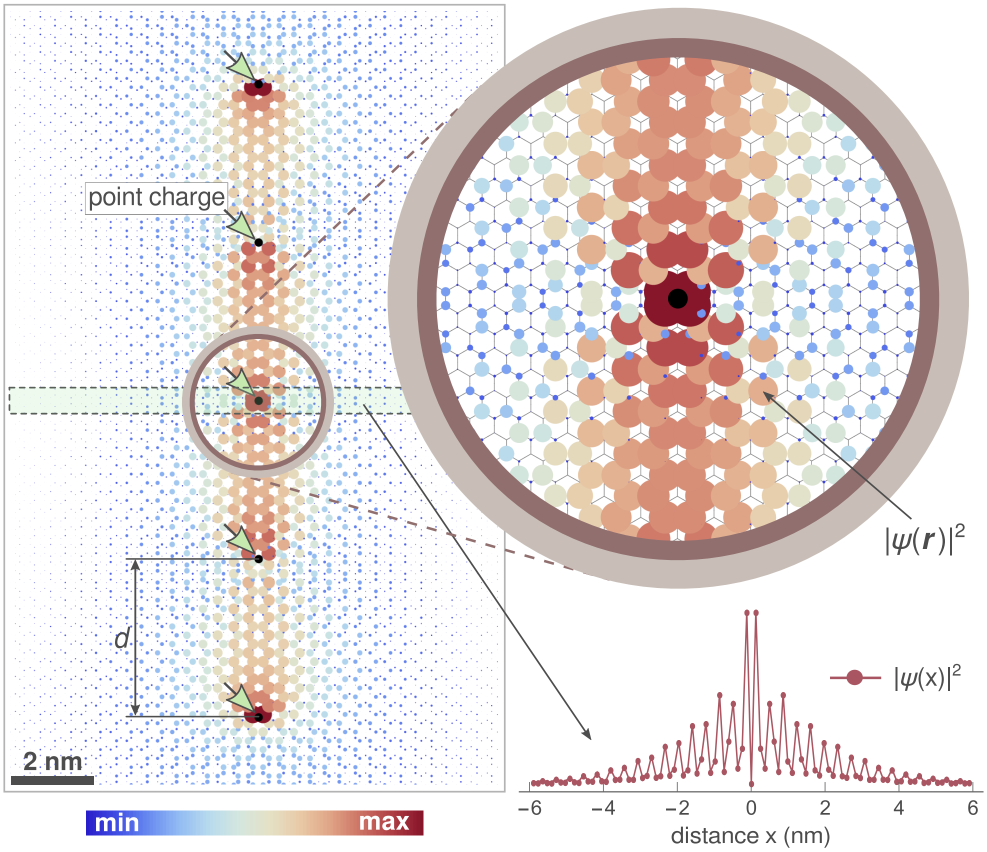

Supplementary Figure 12: Density plot of the wave-function associated with a supercritical resonant state in graphene near the Dirac point obtained from exact diagonalization of the Hamiltonian discussed in Supplementary Note 7 (except for the smaller number of 5 charges, all the parameters are the same as those used in Fig. 2l of the main text: $d = 2a$, $Q = 0.5$ and $\lambda_s = 10\,\text{nm}$). Black dots mark the positions of the Coulomb centers used in the calculation and the colored disks reflect the state's local probability density both through size and color. The charges are separated by $d = 3.8\,\text{nm}$ as in the experimental $2a$ array and the total system has 16,000 carbon atoms spanning $19 \times 21\,\text{nm}^2$ (the image shown is cropped to the central region to emphasize the details of the supercritical wavefunction). The top inset shows a close-up near the central charge where rapid decay is visible against the underlying honeycomb lattice. The bottom inset shows the wave-function cross-section along a line perpendicular to the array (boxed region, cf. Supplementary Figure **11**).

Supplementary Note 11 — Estimation of the effective potential parameters

Our comparison of the simulated and experimental dI/dV curves for both an isolated molecule and the arrays reveals that the best agreement is obtained when the parameters Q and λ_s in the potential (5) take the values

$$Q \approx 0.5, \qquad \lambda_s \approx 10\,\text{nm}. \tag{35}$$

In addition to the data and results shown in the main text for the arrays of different periodicities, Supplementary Figure **13** shows that this value of the coupling strength also provides the best match to the conductance traces characteristic of an isolated molecule in the charged state.

From our earlier definitions, recall that

$$Q \equiv \frac{Ze^2}{\kappa_g \kappa_e tc}, \qquad \kappa_g \equiv 1 + \frac{\pi\alpha_0}{2\kappa_e}, \qquad \alpha_0 \equiv \frac{e^2}{\hbar v_F} \simeq 2.5.$$

Using the parameters $t \simeq 2.7$ eV and $c \simeq 0.142$ nm for graphene, and the effective dielectric constant for graphene at the BN/air interface $\kappa_e = (1 + \kappa_{\text{hBN}})/2 \approx 2.5$ [24, 22, 23], we obtain $\kappa_g \approx 2.6$ for the short-range graphene dielectric contribution within the RPA corrected with the effect of the BN substrate, and

$$Q \approx 0.58\,Z. \tag{36}$$

Therefore, seeing that our best agreement with experiment takes place with $Q \approx 0.5$, this would predict that the effective valence per molecule is approximately

$$Z \approx 0.86. \tag{37}$$

This result is in quite good agreement with the effective valence of F4-TCNQ in the charged state where its LUMO (lowest unoccupied molecular orbital, for the molecule in isolation at equilibrium) is occupied [25]. According to these parameters, the supercritical threshold of an isolated molecule would occur if its effective valence surpasses

$$Z_c \approx 0.75/0.58 = 1.3. \tag{38}$$

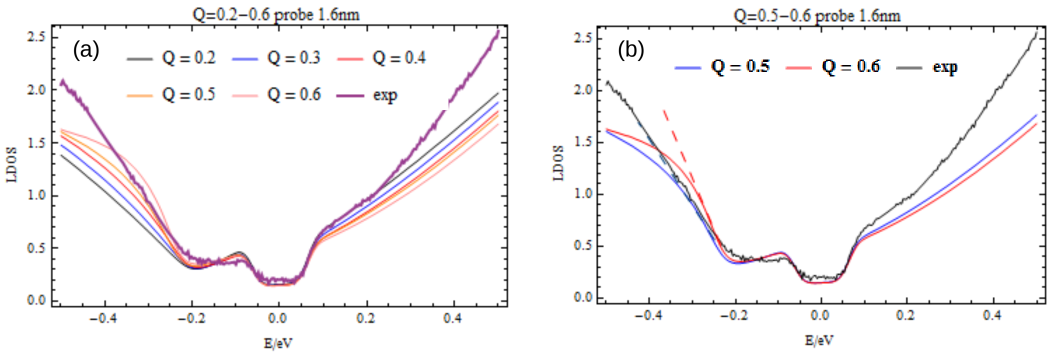

Supplementary Figure 13: Comparison of the measured dI/dV curve taken at a distance of 1.6 nm from an *isolated* charged molecule (labeled "exp") and the corresponding simulated traces with different values of the coupling parameter Q. The Dirac point lies at $V_b \simeq -0.2\,\text{V}$. Panel (a) shows a comparison in the range $0.2 \leq Q \leq 0.6$ while (b) illustrates the criterion used to determine the best Q as that which yields a slope near the Dirac point closest do the experimental dI/dV. In this case, the blue-dashed line ($Q = 0.5$) follows the experimental curve in that region, while the red ($Q = 0.6$) visibly deviates, making the former the adequate choice.

Supplementary Note 12 — Supercritical threshold of an array of subcritical charges

12.1 Scaling arguments

Consider an array of N unscreened charges of valence Z in graphene at positions $\boldsymbol{R}_n = n\,d\,\boldsymbol{u}_y$. Within the Dirac approximation, the electronic Hamiltonian for this problem is

$$H = \hbar v_F\,\boldsymbol{\sigma}\cdot\boldsymbol{\nabla} + \frac{Ze^2}{\kappa}\sum_{n=1}^{N}\frac{1}{|\boldsymbol{r} - nd\boldsymbol{u}_y|}, \tag{39}$$

where κ is the overall dielectric constant. Under a scaling transformation $\boldsymbol{r} \to \Lambda\boldsymbol{r}$, it changes to

$$H = \frac{\hbar v_F}{\Lambda}\,\boldsymbol{\sigma}\cdot\boldsymbol{\nabla} + \frac{Ze^2}{\Lambda\kappa}\sum_{n}\frac{1}{|\boldsymbol{r} - (nd/\Lambda)\boldsymbol{u}_y|}. \tag{40}$$

Notice that the coupling parameter remains the same,

$$g \equiv \frac{Ze^2}{\hbar v_F \kappa} \quad \to \quad \frac{Ze^2\Lambda}{\Lambda\hbar v_F\kappa} = g, \tag{41}$$

whereas the inter-charge distance has changed as $d \to d/\Lambda$. Hence, this scaling transformation induces the transformations

$$H \to \frac{H}{\Lambda}, \qquad d \to \frac{d}{\Lambda}, \qquad g \to g. \tag{42}$$

The scaling of d reflects the intuitive expectation that, when looking at large distances ($\Lambda \gg 1$), the physics of the finite array of N charges should be approximately that of an effective total charge. In fact, when $\Lambda \to \infty$, the potential term becomes

$$\frac{Ze^2}{\kappa} \sum_{n=1}^{N} \frac{1}{|\boldsymbol{r} - (nd/\Lambda)\boldsymbol{u}_y|} \approx \frac{Ze^2}{\kappa r} \sum_{n=1}^{N} = \frac{NZe^2}{\kappa r}, \tag{43}$$

which, indeed, corresponds to the effective Coulomb field due to N charges.

This just shows us that, since the kinetic and potential energy in the low energy description of electrons in graphene has the same length scaling dimension, the physics of an array of N charges seen at distances $r \gtrsim Nd$ is the same as that of a single charge with effective valence NZ at energies scaled down to E/Λ. The supercritical threshold is therefore determined by

$$Z\alpha > \frac{0.5}{N}. \tag{44}$$

A similar conclusion has been obtained by studying approximate solutions of equation (39) in the particular case $N = 2$ by either analyzing the asymptotic behavior of the solutions [26], or solving the problem variationally in a LCAO (linear combination of atomic orbitals) scheme [27].

Although this might seem a somewhat trivial conclusion from the physical standpoint so long as we analyze the problem from the far field, there are important qualitative details that determine the near field and the practical observability of the quasi-bound supercritical states and other supercritical signatures in experiments. Even though the problem with $N > 1$ charges has no exact solution, these scaling properties allow us to discuss its key features on the basis of the exact results known for a single supercritical charge. The most important one for local spectroscopic studies is the fact that the most tightly bound supercritical states are to be localized within the scale of regularization of the Coulomb ($1/r$) field, $r = r_0$. Whereas for a single supercritical impurity that regularization distance is of the order of the lattice parameter ($r_0 \sim c$) [7, 8], in a set of subcritical charges, such distance will be that which encloses the minimum number of charges required to define a supercritical unit. For example, for N charges separated by d with $0.25 < Z < 0.5$, the regularization distance should be simply dictated by $r_0 \sim d$, because each neighboring pair is itself supercritical as a unit. The spread of the supercritical states over larger regions of space compared to the lattice constant implies a weakening of their signatures in local tunneling spectroscopy, simply because the normalizability constraint redistributes the local probability density over larger areas, and that implies they contribute less to enhancing the LDOS. Therefore, even though a set of impurities might be nominally supercritical and the associated quasi-localized

states contribute in a non-trivial way to the local screening of the charges, these might not be as sharply detectable in STM measurements. This perspective explains the progressive reduction in the intensity of the resonant peak in the experimental dI/dV traces shown in Fig. 2(e–h) (main text) with increasing period d of the molecular array.

In an experimental setting, one has to compound these arguments with the fact that in any doped system the Coulomb field will be screened at large distances. On the one hand, screening affects whether or not the far-field, defined as the regions where the potential is approximately $NZe^2/\kappa r$, exists or not. Obviously, the supercritical regime of the array should disappear whenever $d \gg \lambda_s$ since, then, the combined potential of any pair of different charges can never be of the Coulomb form. In addition, according to the arguments above, the tightest supercritical states are pushed out so that they spread to distances that are also $\sim d$ away from the array. In order for these states to remain in the presence of screening, one must have at least $\lambda_s \gtrsim d$ to ensure they still constitute a solution of the wave equation in all the regions $r \lesssim \lambda_s$. Furthermore, in contrast to the unscreened case where there is, formally, a large number of resonant supercritical states "localized" within increasingly outer regions of space, a finite λ_s necessarily implies a finite number of such states since they cannot be supported when their natural localization radius is larger than the screening distance. Direct numerical confirmation of this suppression of the supercritical regime with increasing screening is discussed in the next section.

12.2 Numerical tests of the supercritical threshold in arrays of charge

12.2.1 Diving of energy levels

Graphene has a gapless electronic spectrum. For simplicity, consider for the moment a single Coulomb charge. Unlike the corresponding situation of interest in the QED of heavy ions (or gapped graphene) where an increase in the strength of the Coulomb field progressively lowers the bound state energies until they merge with the lower (positron or hole) continuum [30, 31]—at which point the supercritical threshold occurs—, in graphene the supercritical threshold is much more abrupt. First, the modifications of the spectrum in the presence of the field are perturbative without anomalous features (e.g. resonances) up to $Z\alpha = 0.5$ and the transition to the supercritical regime is a non-perturbative effect [7, 8, 21, 11]. Second, quasi-bound resonant states appear for $Z\alpha > 0.5$ and, in strict terms, the solutions of the single particle equations lead to the "instantaneous" emergence of an infinite number of quasi-bound states with resonant energies that accumulate at the Dirac point [7, 9]. This is in contrast with the corresponding problem in the presence of a gap, where these states develop incrementally [28] (from this perspective, the supercritical regime of graphene is equivalent to the ultra-relativistic limit in the corresponding problem in QED because the Coulomb energy is much larger than mc^2). This fact has non-trivial consequences for the self-consistent screening of the charge that these states induce in graphene [8].

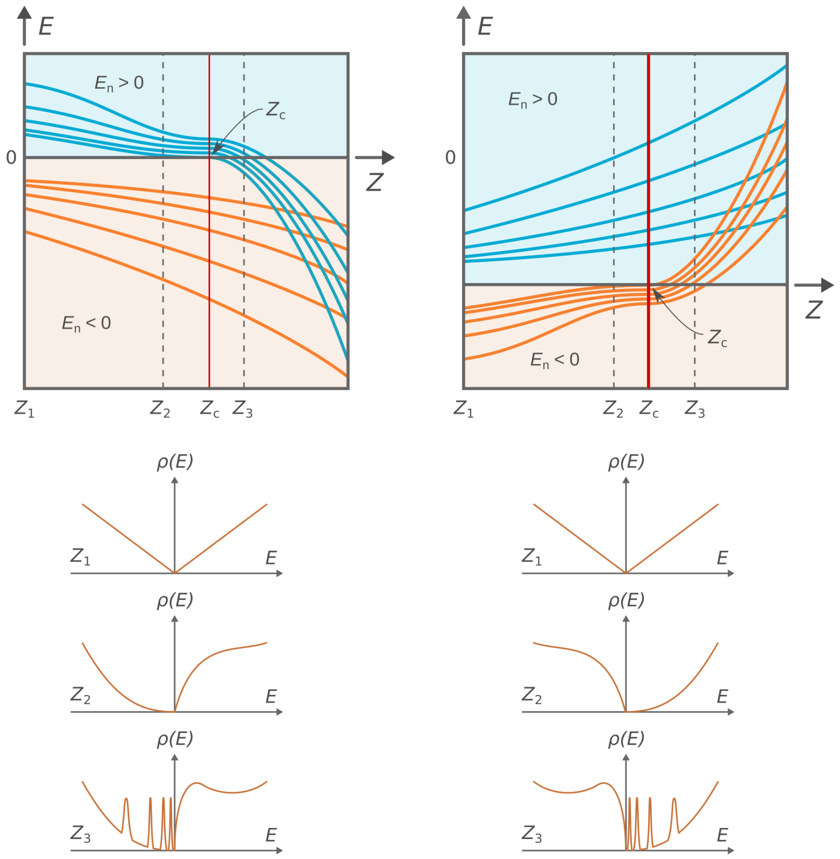

Supplementary Figure 14: Representation of the difference between the electronic spectrum below and above the supercritical threshold of a *single* Coulomb center in graphene. Left column (attractive case): if $Z < Z_c$, the positive energy levels $E_n > 0$ accumulate near $E \gtrsim 0$ while negative energy levels become more sparse around $E \lesssim 0$ (top panel); beyond $Z > Z_c$ a number of positive energy levels incrementally dives into the region $E < 0$; all such states are called supercritical. The lower panels depict the LDOS at representative Z below and above the supercritical threshold, including the appearance of sharp resonances associated with the quasi-localized supercritical states above Z_c. Right column (repulsive case): the same as the previous case, except that now particles ($E > 0$) and holes ($E < 0$) have their roles and behavior with increasing Z exchanged. Namely, the diving occurs now for negative energy states that penetrate the positive energy continuum above Z_c. The repulsive case is the one relevant for our charged F4-TCNQ molecules which have an extra electron occupying the LUMO and, hence, act as repulsive charges to electrons.

In the absence of an analytical solution, one procedure to assess whether a set of charges is supercritical or not consists in tracking any states crossing the Dirac point ($E_D = 0$). Without external potentials, there is a particle-hole symmetry in the low energy spectrum of graphene which is captured by our tight-binding model 1 as well as in the Dirac approximation to it close to E_D. Since the tight-binding Hamiltonian contains only one (the relevant) band, our model has an explicitly finite bandwidth ($-3t \leq E \leq +3t$) and the number of states above and below E_D is precisely the same. Any calculation of the exact spectrum will reflect that, and it helps to think of the spectrum of a finite (though large) system where we can track each energy level. When the Coulomb field of one charge is added, all levels will be perturbed, namely, moving downwards (upwards) in energy if the field is attractive (repulsive). However, so long as $Z\alpha < 0.5$, levels close to E_D cannot cross this point so that they, instead, accumulate on the positive or negative side of the Dirac point depending on the sign of the potential (in fact, the behavior under a given external potential can be obtained directly from that of the same potential with opposite sign with the substitution $E \to -E$.). This can be established exactly in the Dirac approximation [7, Fig. 1(c)] and is confirmed by inspection of the exact numerical spectrum in the tight-binding model [28, 29].

At the supercritical threshold $Z\alpha = 0.5$ a number of things changes in a non-trivial way, one of them being that the energy levels can now cross $E = 0$ as the potential strength is further increased. So, if the potential is attractive, above this threshold a number of electron-like states (i.e., those having $E > 0$ at zero or subcritical field) will have crossed to the hole-like region ($E < 0$), the system remaining gapless all along (see Supplementary Figure **14** for the case of a *single* Coulomb center). This *diving* of electron-like states into the hole continuum is entirely analogous to the process that happens when there is a finite gap [28] and these states are those responsible for the supercritical resonances in the LDOS. A consequence of immediate practical relevance is that, above the supercritical threshold, the number of states below and above E_D is no longer the same when we analyze the spectrum of a finite (though large) system. Therefore, identifying the onset of this "diving" of levels in the opposite continuum provides a means to identify the supercritical threshold in a situation where and analytical result is not available.

Supplementary Figure **15** shows a number of representative examples of the application of this criterion to identify whether a multi-charge system is super or subcritical depending on the valence per charge Z, their separation d, as well as the impact of having a finite screening length λ_s in the Coulomb field. While the panels in Supplementary Figure **15** summarize each case in terms of the number of levels that have penetrated into the positive energy continuum, the respective panels in Supplementary Figure **16** show the detailed evolution of E_n for each case, in the vicinity of the Dirac point.

First, we exemplify this phenomenon for a single unscreened charge (panel a), where it is clear that the threshold occurs for $0.7 < Q < 0.8$, consistent with the exact $Q_c = 0.75$. In panel (b) we have an array of 5 unscreened charges and show how the criterion is consistent with the expectation

that the supercritical threshold should occur at $Q > Q_c/N = 0.15$, along the lines discussed earlier. A single charge that is nominally supercritical ($Q = 2.0$) but has a screened Coulomb field ceases to show level diving when $\lambda_s \lesssim 1.25c$ (panel c), consistent with the fact that the dominant supercritical states are localized within a region of size $\sim c$ in this case, and are thus resilient to screening of the potential up to very small λ_s. In contrast, an array of screened charges ($Q = 0.5$, $N = 5$, $d = 27c$, corresponding to the experimental case "$2a$") ceases to show supercritical behavior at rather larger screening lengths, $\lambda_s \sim 20c \sim d$ (panel d), which is in line with our earlier discussion based on the scaling properties of the Dirac equation. Finally, in panel (e) we show that if the coupling strength for an array of $N = 3$ unscreened charges is held constant at $Q = 0.2$ or $Q = 0.5$ while their separation is varied, for $Q = 0.2$ no diving of levels occurs (as we would expect because $Q < Q_c/3$), while if $Q = 0.5$, the system is always supercritical irrespective of the separation between the charges. Note that in this analysis the actual number of states that have dived is not significant *per se*, but rather whether that number is zero or finite because, as we have an explicitly finite system in our exact diagonalization calculations, that number depends on the system size. But, as these different examples sharply demonstrate, whether at least one is seen to cut across E_D or not provides a robust identification of the threshold.

The cases shown in Supplementary Figures **15** and **16** also directly confirm the conclusions advanced in the previous section regarding the supercritical threshold as a function of the number of charges, separation and screening on the basis of scaling arguments.

12.2.2 Spread of supercritical states for a pair of charges

Our scaling argument above suggests that any set of N subcritical charges which, as a whole, exceeds $NZ > Z_c$ will be supercritical, and that the typical localization length of the dominant (most tightly bound) supercritical state is of the order of the inter-charge separation d (since d is the effective regularization distance of the potential $\sim NZe^2/\kappa r$ in the far-field). We have confirmed this by direct inspection of the spatial spread of the wave functions obtained from the exact diagonalization of a pair of unscreened and subcritical charges as a function of their separation. Representative cases are shown in Supplementary Figure **17** (the case of $N = 5$ charges is shown in Fig. 4 of the main text). Note that, since the state must remain normalized, the expansion of the area around the charges it encompasses as d increases implies a decrease of the local probability amplitude at any given site. From the point of view of an STM experiment, this spreading of the wave function extension, translates into less tunneling current. As a result, even if the supercritical states are present, their signature in dI/dV should become progressively weaker as charges are moved farther apart and these states, correspondingly, spread over larger and larger scales.

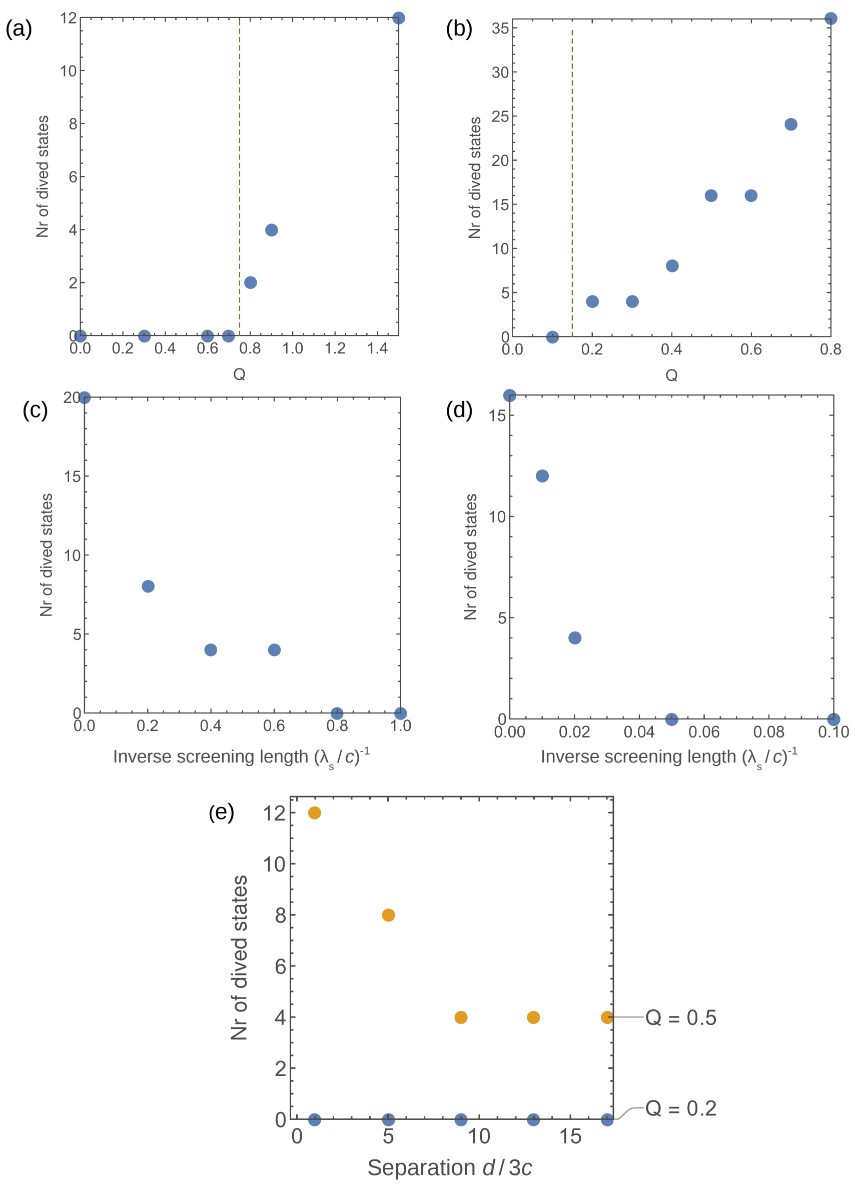

Supplementary Figure 15: Onset of the supercritical regime identified numerically by the crossing of negative energy levels over the Dirac point ($E = 0$). (a) For a single unscreened charge, no levels dive as long as $Q < Q_c = 0.75$ [cf. equation (8)], which is expected because Q_c marks the supercritical threshold. (b) An array of 5 unscreened charges is expected to be supercritical at $Q > Q_c/N = 0.15$. The energy levels are seen to begin diving precisely beyond that value. (c) A screened single charge ($Q = 2.0$) ceases to show level diving when $\lambda_s \lesssim 1.25c$ and, hence, the supercritical regime is somewhat robust to screening up to λ_s of the order of the lattice spacing c (d) An array of screened charges ($Q = 0.5$, $N = 5$, $d = 27c$, corresponding to the experimental case "2a") ceases to show supercritical behavior at rather larger screening lengths, $\lambda_s \sim 20c \sim d$. (e) The coupling strength is held constant at $Q = 0.2$ and $Q = 0.5$ for an array of $N = 3$ unscreened charges while their separation is varied in units of $3c$. As expected, for $Q = 0.2$ no diving of levels occurs because $Q < Q_c/3$. On the other hand, when $Q = 0.5$, the system is always supercritical irrespective of the separation between the charges. All panels based on results of exact diagonalization with 160×100 atoms.

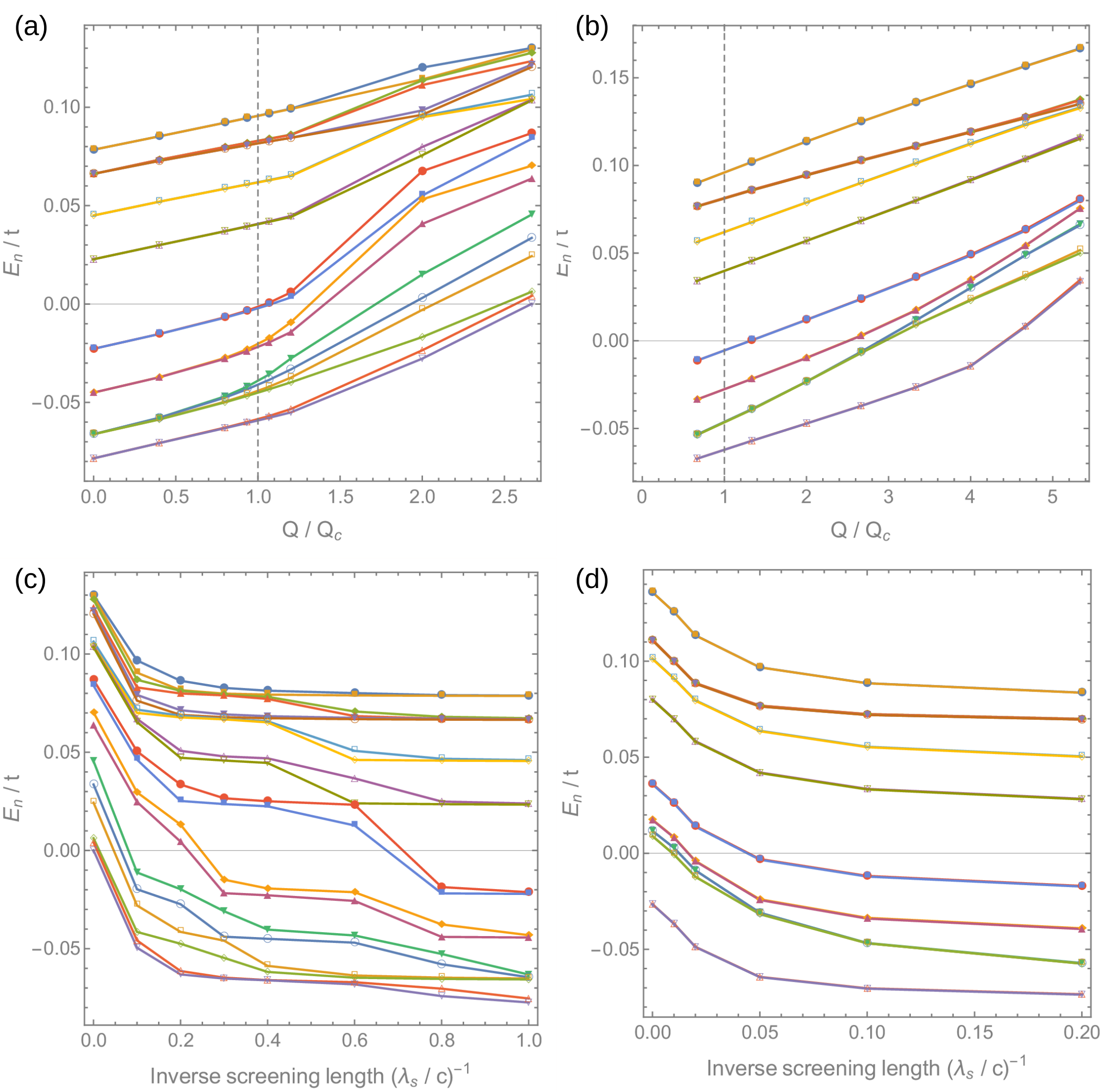

Supplementary Figure 16: Evolution of the energy levels determined by exact diagonalization on the finite-sized systems ($N = 160 \times 100$ atoms) discussed in panels (a) to (d) of Supplementary Figure 15, respectively. The "diving" phenomenon corresponds here to a negative energy level crossing $E = 0$ into the positive side (the Coulomb centers are positively charged) as the potential strength is varied, either through changing Q or the screening length λ_s. Only the 20 levels closest to the Dirac point of pristine graphene are shown. Refer to the caption of Supplementary Figure 15 for details and parameters utilized in each panel.

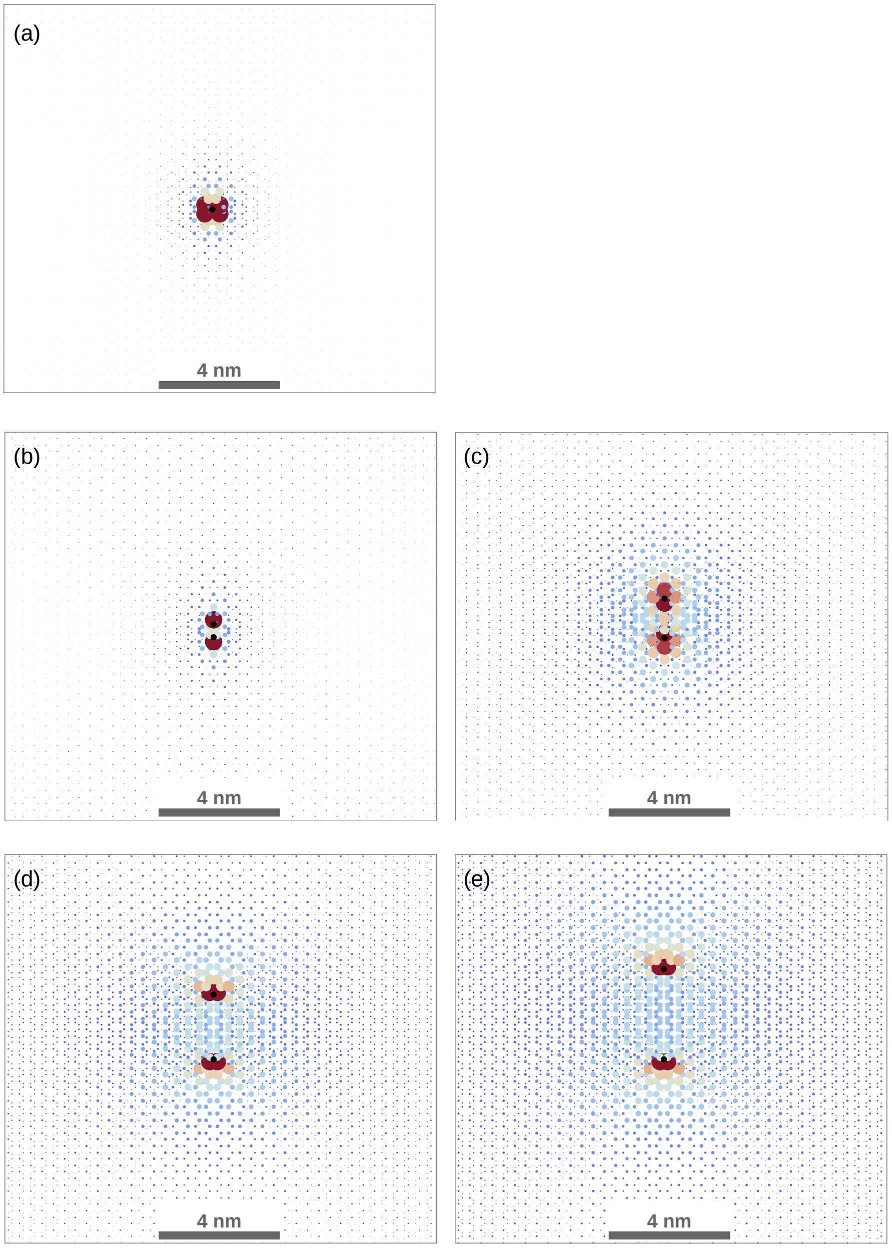

Supplementary Figure 17: Eigenfunction of the most bound supercritical state of a pair of charges as a function of charge separation (unscreened potential with $Q = 0.6$; only a close-up of the central region of our 160×100 atom system is shown for clarity). Black dots mark the positions of the point charges and the size of the colored disks is proportional the local probability density. (a) $d = 0$ nm ($E = 0.067t$), (b) $d = 3c \simeq 0.4$ nm ($E = 0.054t$), (c) $d = 9c \simeq 1.3$ nm ($E = 0.015t$), (d) $d = 15c \simeq 2.1$ nm ($E = 0.013t$), (e) $d = 21c \simeq 3.0$ nm ($E = 0.011t$). Notice how the spatial extent of the wave function is similar in (a) and (b) but increasing the charge separation d makes the wave function spread over increasingly large areas, with a characteristic linear dimension that is roughly of the same order of d.

12.3 Beyond the individual supercritical threshold

Although this case is not directly relevant for our current experiments, for completeness of the physical picture, imagine that one continuously increases the charge/valence on each molecule (Z). It is well understood that, as soon as the supercritical threshold is overcome ($Z\alpha > 0.5$), each charge individually sustains an atomic-like series of resonant states whose respective wavefunctions are now localized within distances of the order of graphene's lattice constant [7, 8, 9]. When such charges are arranged periodically, as in our experimental arrays, one will observe the formation of supercritical energy bands, in the same way that true atomic states lead to energy bands when they hybridize in a conventional crystal. In other words, supercritical bands emerge immediately after the individual supercritical threshold.

Experimentally, this would translate in distinctive behavior in comparison with our current regime where each charge is individually sub-critical:

1. there would appear supercritical states with a localization length smaller than the current one which is essentially dictated by the inter-charge separation;
2. unlike what we see in Figs. 2 of the main text, where the sharp resonance fades with increasing separation, sharp resonant levels should be detected independently of the inter-charge distance d (although their energy will vary with d);
3. the fact that bands of supercritical states emerge beyond the threshold implies a relation between the characteristic width of the supercritical features in dI/dV and the width of the supercritical bands. Since that bandwidth decreases for larger inter-charge separations, the corresponding dI/dV features would become sharper with increasing d.

Hence, if one is capable of driving each charge individually across the supercritical threshold ($Z\alpha > 0.5$), these qualitative features in contrast with the subcritical behavior would altogether allow the experimental identification of the crossover.

Supplementary Note 13 — Effective radial potentials

13.1 Semiclassical radial potential in graphene

Here we consider the semi-classical picture of a *point, unscreened* Coulomb charge in graphene.

Definitions The quantum-mechanical effective Dirac Hamiltonian appropriate for graphene in the absence of any potential is

$$H \equiv v_F \boldsymbol{P} \cdot \boldsymbol{\sigma}, \tag{45}$$

where v_F is the Fermi velocity introduced earlier, $\boldsymbol{P}$ the momentum operator, and $\boldsymbol{\sigma}$ the Pauli matrix vector [32]. The associated energy dispersion reads

$$E_{\boldsymbol{k}} = \pm \hbar v_F K. \tag{46}$$

The "classical" Hamiltonian of a graphene quasi-particle in the presence of a Coulomb field will be written as [9]

$$H(\boldsymbol{R}, \boldsymbol{P}) \equiv \eta v_F P + V(R), \qquad V(R) \equiv \frac{Ze^2}{\kappa R}, \tag{47}$$

where κ represents the overall static dielectric constant and $\eta = \pm 1$ for electrons and holes, respectively (note that this definition implies that $Z < 0$ for a positive Coulomb impurity, and $Z > 0$ for a negative one). In this equation,

$$P^2 \equiv P_x^2 + P_y^2 = P_r^2 + \frac{P_\theta^2}{R^2}, \qquad R^2 \equiv X^2 + Y^2. \tag{48}$$

It is convenient to define the following dimensionless quantities:

$$\varepsilon \equiv \frac{Ec}{\hbar v_F}, \qquad p \equiv \frac{Pc}{\hbar}, \qquad r \equiv \frac{R}{c}, \qquad g \equiv \frac{Ze^2}{\kappa \hbar v_F}, \qquad v(r) \equiv \frac{c}{\hbar v_F} V(R) = \frac{g}{r}, \tag{49}$$

where c is a characteristic length scale which we can take as the C-C distance in graphene ($c \simeq 0.142$ nm).

The dimensionless Hamiltonian hence becomes

$$h \equiv \frac{Hc}{\hbar v_F} = \eta p + \frac{g}{r}. \tag{50}$$

We further recall the effective "fine structure constant" in graphene

$$\alpha \equiv \frac{e^2}{\kappa \hbar v_F}, \qquad g = Z\alpha, \qquad \alpha_{\text{vac}} \equiv \frac{e^2}{\hbar v_F} \simeq 2.5 \quad [t = 2.7\,\text{eV}], \tag{51}$$

and the threshold valence to establish the supercritical regime [7, 8, 11]:

$$g_c \equiv \frac{1}{2} \qquad \longrightarrow \qquad Z_c \equiv \frac{1}{2\alpha}. \tag{52}$$

We now analyze some important qualitative features of the Hamiltonian (50) at the semi-classical level.

Effective radial potential Due to the circular symmetry, the angular momentum is conserved and it is sufficient to analyze the radial problem for fixed angular momentum. Hence we set $p_\theta = l$ and re-write (50) in polar coordinates:

$$h = \eta\sqrt{p_r^2 + \frac{l^2}{r^2}} + v(r). \tag{53}$$

Given an energy ε, the radial momentum reads

$$p_r^2(r) = \left[\varepsilon - v(r)\right]^2 - \frac{l^2}{r^2} = \varepsilon^2 - u_{eff}(r), \tag{54}$$

with

$$u_{eff}(r) \equiv \frac{l^2}{r^2} - v^2 + 2\varepsilon v = \frac{2g\varepsilon}{r} + \frac{l^2 - g^2}{r^2}. \tag{55}$$

This defines the simplified semi-classical effective radial potential $u_{eff}(r)$.

We note in passing that the rigorous way to obtain this effective potential is to express the wave equation associated with the Hamiltonian (47) as an effective 1D Schrödinger equation that, for a general radial potential, can be seen to read

$$\psi''(r) + \left[\varepsilon^2 - u_{eff}(r)\right]\psi(r) = 0, \tag{56}$$

with

$$u_{eff}(r) = -v^2 + 2\varepsilon v + \frac{j(j-1)}{r^2} + \frac{v''}{2(\varepsilon - v)} + \frac{3}{4}\left(\frac{v'}{\varepsilon - v}\right)^2 + \frac{v'}{\varepsilon - v}\frac{j}{r}. \tag{57}$$

(refer to reference [10] for details). Comparison of (55) with this result shows that the former amounts to neglecting the terms involving derivatives of the potential ("spin-orbit" terms), and identifying $l^2 \leftrightarrow j(j-1)$. The crucial element is the presence of a term in u_{eff} that diverges with $(l^2 - g^2)/r^2$, which is entirely captured in the simpler form of the potential (55).

Falling orbits We can see that, if $g\varepsilon > 0$, the effective potential is asymptotically repulsive when $r \to \infty$, whereas the behavior as $r \to 0$ is determined by the relative magnitude of l and g. If the angular momentum is large enough that $|l| > |g|$, the radial potential is everywhere repulsive. In contrast, when $|l| < |g|$, the potential is singularly attractive as $r \to 0$, implying that the semi-classical orbits all fall towards $r = 0$. This is the supercritical regime. Since the angular momentum is conserved, in this semi-classical formulation we are in the supercritical regime if $g > g_c = |l|$ for a given value of l (in the quantum-mechanical treatment, l is replaced by the quantum number associated with the total angular momentum, j, which is made out of orbital and pseudo-spin contributions; as $\min|j| = 1/2$, $g = 1/2$ becomes the absolute supercritical threshold [10]).

This situation is qualitatively illustrated in Supplementary Figure **18**, where we plot the square of

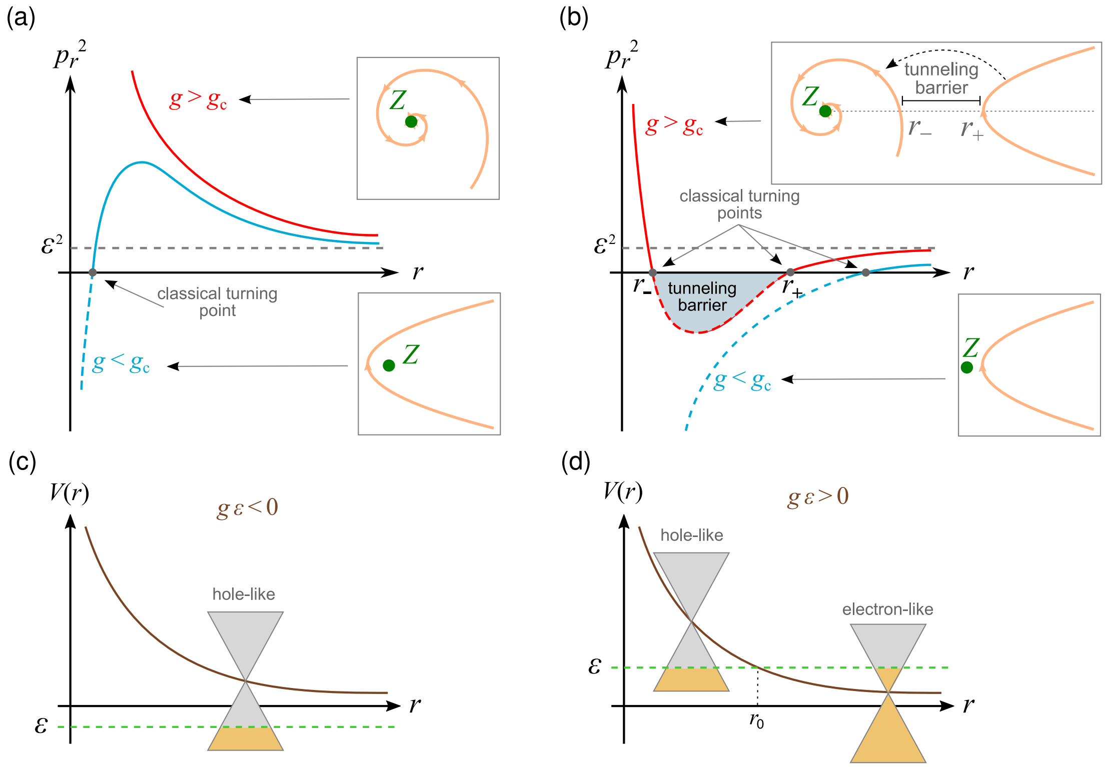

Supplementary Figure 18: Illustration of the semi-classical radial motion of a Dirac particle in graphene under the influence of a point Coulomb field according to eq. (54). (a) Semi-classical radial momentum for $g\varepsilon < 0$, which corresponds to hole states when the Coulomb center is negatively charged, as happens with F4-TCNQ. (b) Semi-classical radial momentum when $g\varepsilon > 0$. If $g > g_c$, there is a finite region, $r_- < r < r_+$, that is classically forbidden; it defines a quantum-mechanical tunneling barrier that allows trapping the Dirac quasiparticles into quasi-localized (resonant) states near the charge. (c) At negative energies, the graphene quasi-particles have a hole character. (d) At positive energies, they are electron-like in the regions of space $r > r_0 \equiv g/\varepsilon$ and hole-like closer to the charge.

the semi-classical radial momentum, p_r^2, defined in eq. (54). The panels in the left (right) column pertain to cases where $g\varepsilon > 0$ ($g\varepsilon < 0$). To be specific, since in our system the F4-TCNQ molecules are negatively charged (hence, $g > 0$ according to our convention above), $g\varepsilon > 0$ applies to the positive energy states (electrons), and $g\varepsilon < 0$ applies to holes.

Since p_r^2 must be a non-negative quantity, there will be classical turning points at the radial positions where $p_r(r)^2 = 0$. This allows one to directly identify the qualitative features of the particle's orbit from the plots shown in Supplementary Figure **18**. In panel (a), which reflects the situation of a hole, we see that there are two possibilities: if $g < g_c = l$, the trajectories have a finite periapsis (minimum distance of approach) given by $r_{\min} = \frac{|l|-|g|}{|\varepsilon|}$, but subsequently move towards infinity; on the other hand, if $g > g_c = l$, the particle falls towards $r = 0$. The insets of Supplementary Figure **18**(a) show the schematic orbits in each case. Hence, as intuitively ex-

pected, a hole is always attracted towards a negative charge, and might collapse or not depending on the magnitude of g. In either case, there are only open orbits, and there is at most one turning point.

The situation is qualitatively different in the case of an electron state ($\varepsilon > 0$) that propagates from infinity towards the proximity of the charge, as depicted in panel (b). In this case, if $g < g_c$ there is only a turning point indicating the periapsis of the open hyperbolic orbit; this is the intuitively expected scenario because of the repulsion that is felt by an electron near a negative charge. However, if $g > g_c$, there are two turning points defining two classically allowed regions [9]:

$$r < r_- \equiv \frac{|g| - |l|}{|\varepsilon|}, \qquad \text{and} \qquad r > r_+ \equiv \frac{|g| + |l|}{|\varepsilon|}. \tag{58}$$

From the shape of p_r^2 in each region, we see that r_+ constitutes a minimum distance of approach for a particle in the region $r > r_+$, consistent with the fact that an electron is repelled by a negative charge and hence has a finite periapsis. But, in contrast, in the region $r < r_-$ the particle falls onto the charge. In order to reconcile the apparently contradictory behavior in the latter case (that suggests attraction, rather than repulsion) we must realize that, in this situation, the character of the particle is electron-like in the region $r > r_+$, but hole-like for $r < r_-$. This is illustrated in panel (d) that shows the relative position of the particle's energy and the Dirac point in the two different regions.

Supercritical states in the quantum-mechanical treatment We note that only when $g\varepsilon > 0$ and $g > g_c$ do we have a classically forbidden region: $r_- < r < r_+$. In the quantum-mechanical treatment of the problem, this region defines a finite tunneling barrier that allows electrons beyond r_+ to tunnel as holes into the inner region $r < r_-$ where they can reside over a large lifetime [9]. The typical localization length of these states is defined by r_-. This translates in the appearance of strong resonances in the local density of states [7, 8, 29] or, equivalently, by the existence of eigenstates strongly localized in the vicinity of the Coulomb charge [cf. Supplementary Figure **17**(a)]. Such states are detectable in STM experiments by marked resonances in dI/dV spectra [24].

An interesting feature of the effective potential in eq. (55) is that it depends on the particle's energy ε. One consequence for this problem is that, in the supercritical regime where $g\varepsilon > 0$ and $g > g_c$, the tunneling barrier cannot be avoided at higher energies because the maximum of $u_{eff}(r)$ occurs at

$$r_e = \frac{g^2 - l^2}{g\varepsilon}, \qquad \text{and} \qquad u_{eff}(r_e) = \frac{\varepsilon^2}{1 - l^2/g^2} > \varepsilon^2.$$

According to eq. (54), this implies that there is always a classically forbidden region irrespective of the value of the particle's energy. In other words, the particle sees a potential barrier at all

energies. One consequence of this is the existence of an infinite number of resonant states that accumulate towards $\varepsilon \to 0$ in the quantum-mechanical treatment of the problem [7, 8, 9]. Since eq. (58) implies that particles with smaller energy are confined over larger spatial regions (because $r_- \propto 1/\varepsilon$), the resonant states spread further and further away from the potential source as $\varepsilon \to 0$. In addition, the fact that there is no natural length scale in the Hamiltonian (50) also implies that one can have supercritical states localized at arbitrarily small distances from the potential origin down to the atomic scale [8]; this is precisely the situation seen for the exact eigenfunction plotted in Supplementary Figure **17**(a) that shows the most tightly-bound exact wave function in the tight-binding model for a point supercritical charge.

A screened Coulomb center As indicated at the start, the above discussion applies for a bare Coulomb potential. Since the essence of the supercritical regime is the development of tightly localized resonant states, one expects *a priori* that states with localization length smaller than the screening radius will be largely unaffected by the screening of the potential. Hence, supercritical physics is expected to persist in the presence of screening to the extent we now describe. For the remainder, we focus on the situation $g\varepsilon > 0$ and $g > g_c$ which, as seen above, is the appropriate one in relation to the quasi-localized states.

To be more specific, a screened Coulomb center can be discussed in a generic way by introducing a screening function $F(r)$, similar to that introduced in the context of eq. (26), which is continuous, has the asymptotic properties

$$F(0) = 1, \quad F(\infty) = 0, \tag{59}$$

and a typical screening length λ_s at which $F(r)$ crosses over between these two asymptotic values.

Quite generically, as long as λ_s is much larger than all the characteristic classical turning points discussed above, the qualitative supercritical behavior will persist. Namely, the classically-forbidden region will remain, and so will the quasi-localized resonant states. More specifically, since (58) shows that the turning points depend explicitly on the reciprocal energy, screening affects those supercritical states that would spread (in the absence of screening) over distances $r \gtrsim \lambda_S$, but not those localized within $r \lesssim \lambda_S$. This follows because the supercritical physics is a short-distance feature that results from the "opening" of a classically allowed region close to the impurity ($r < r_-$) and, therefore, it is reasonable that these short-distance details will not change if λ_s is large.

In the general case of an arbitrary, and possibly small, λ_s, we can generalize the discussion above in the context of the bare Coulomb problem by replacing the Coulomb potential

$$v(r) = \frac{g}{r} \longrightarrow \frac{g\,F(r)}{r} \tag{60}$$

in eqs. (54) and (55). The classically forbidden region exists between the turning points that solve

$$p_r^2(r) = 0 \qquad \longrightarrow \qquad r_\pm : \quad r^2\varepsilon^2 - 2rg\varepsilon F(r) + g^2 F^2(r) - l^2 = 0, \tag{61}$$

which, we can rearrange as

$$r_\pm : \quad F(r) = \frac{\varepsilon}{g} r \pm \frac{|l|}{|g|}. \tag{62}$$

Graphical inspection shows that this equation always admits two solutions when $|g| > |l|$ and $F(r)$ is a smooth function with the property (59). Therefore, irrespective of how strongly one screens the Coulomb potential (i.e., of how small λ_s is), there remains a classically-allowed region close to the potential source, $r < r_-$, where all the orbits fall onto the origin (this is an intuitive outcome because a screening function like (59) will never remove the $1/r^2$ singularity). However, unlike the bare Coulomb case, the potential barrier will no longer be present at all energies. In particular, it disappears for energies smaller than $\sim g/\lambda_s$ which results in a finite number of resonant states, as opposed to their infinite number in the unscreened case. Since the localization length increases with decreasing energy, screening affects the quasi-localized states that are less tightly bound, as indicated above, suppressing those having a localization length larger than λ_s. But, because the most tightly bound states are localized within a region of the order of graphene's unit cell [7, 8], it follows that (some) supercritical resonant states will remain in the presence of screening, provided λ_s is not so small that it becomes of the order of graphene's lattice constant. In other words, even though we don't have anymore an infinite number of supercritical states extending arbitrarily far from the potential source, there remain supercritical states with a localization length smaller than the screening radius of the potential.

13.2 Effective Schwarzschild geodesic potential

We consider here the standard discussion of the motion of *massless* particles under the influence of *only* the gravitational field (free-fall) in general relativity, as discussed in standard textbooks such as Hartle's [33]. We will consider the convention of using the signature $(-1, +1, +1, +1)$ for the flat-space metric. In this section, c is the speed of light and we do not consider any other scenario than *static* mass distributions.

Geodesic equations Free-falling massive test particles move along space-time geodesic curves. The geodesic equation is given by

$$\frac{d^2x^\alpha}{d\tau^2} = -\Gamma^\alpha_{\beta\gamma}\frac{dx^\beta}{d\tau}\frac{dx^\gamma}{d\tau}, \tag{63}$$

where x^α, $\alpha \in \{0, 1, 2, 3\}$ are the space-time coordinates of the test particle, τ is the particle's proper time,

$$d\tau^2 = g_{\alpha\beta} dx^\alpha dx^\beta, \tag{64}$$

and $\Gamma^\alpha_{\beta\gamma}$ are the Christoffel symbols that derive directly from the metric $g_{\alpha\beta}$ and its derivatives [33]. For example, in flat space $\Gamma^\alpha_{\beta\gamma} = 0$ and, if we consider the non-relativistic limit where $v \ll c$, then $\tau \approx t$ and the geodesic equation becomes

$$\frac{d^2 x^\alpha}{dt^2} = 0, \tag{65}$$

which is precisely Newton's equation of motion in the absence of forces (flat-space implies no gravity). Formally, determining the free-fall motion of massive particles (i.e. their orbits in space-time) under an arbitrary gravitational field, reduces to integrating the geodesic equation (63) once the metric tensor associated with the mass-energy distribution is known (which, in turn, follows from the solution of Einstein's field equations).

The trajectory of light rays through space-time is governed by an equation formally similar to (63), except that the proper time can no longer be used as the affine parameter that describes the orbits because $d\tau = 0$ for light. In this case, the geodesic motion of light rays is described by

$$\frac{d^2 x^\alpha}{d\lambda^2} = -\Gamma^\alpha_{\beta\gamma} \frac{dx^\beta}{d\lambda} \frac{dx^\gamma}{d\lambda}, \tag{66}$$

subject to the null condition

$$g_{\alpha\beta} \frac{dx^\alpha}{d\lambda} \frac{dx^\beta}{d\lambda} = 0. \tag{67}$$

Schwarzschild geometry outside a spherical star The outer solution to Einstein's equations in the presence of a static and spherically symmetric mass distribution (a star, for example) has been obtained by Schwarzschild. The metric reads, in the coordinates (t, r, θ, ϕ),

$$ds^2 = -\left(1 - \frac{2M}{r}\right) dt^2 + \left(1 - \frac{2M}{r}\right)^{-1} dr^2 + r^2 d\theta^2 + r^2 \sin^2\theta \, d\phi^2, \tag{68}$$

where M is the total mass of the star. The fact that this is the *outer* solution/metric means that if the mass distribution is within a region $r < R$, it applies only in the exterior region $r > R$. This equation is written in geometrized units $G = c = 1$, where G is Newton's gravitational constant. To reinstate the conventional units, one replaces

$$M \to \frac{GM}{c^2}, \quad t \to ct, \quad \text{etc.} \tag{69}$$

The Schwarzschild metric has two important symmetries: it is independent of time, which means

that it is invariant under displacements along the Killing vector

$$\boldsymbol{\xi} \equiv (1,0,0,0), \tag{70}$$

and it is also independent of ϕ, which likewise implies invariance under another Killing vector

$$\boldsymbol{\eta} \equiv (0,0,0,1). \tag{71}$$

These two symmetries reduce, respectively, to conservation of the total energy and angular momentum in the non-relativistic limit, and arise from the static nature and spherical symmetry of the mass distribution. Their conservation under geodesic (free-fall) motion can be characterized by stating that [33]

$$e \equiv -\boldsymbol{\xi} \cdot \boldsymbol{u}, \qquad \ell \equiv \boldsymbol{\eta} \cdot \boldsymbol{u}, \tag{72}$$

are constant along geodesics, where $\boldsymbol{u}$ is the 4-velocity

$$u^\alpha \equiv \frac{dx^\alpha}{d\tau}. \tag{73}$$

Written in these so-called Schwarzschild coordinates, the metric (68) has a singularity at the radius

$$R_{\text{sc}} \equiv 2M, \tag{74}$$

(or $2GM/c^2$ in conventional units) which is called *Schwarzschild radius.* A star always has R_{sc} inside its surface. Otherwise, the massive body is a black hole.

Effective geodesic potentials To analyze the trajectory of particles or light in the vicinity of a spherically symmetric mass distribution one needs to solve the geodesic equations (63) or (66) for the metric (68). In the process of doing so, it is illuminating to write the resulting equations in a form that is familiar from Newtonian physics. In particular, whenever we're faced with a problem with spherical symmetry in Newtonian physics, one uses the conservation laws of energy and angular momentum to reduce the analysis to the radial motion. This leaves us with solving a single equation of motion for the radial coordinate in terms of an effective radial potential (precisely as was done in the previous section when discussing the semi-classical features of the Coulomb problem in graphene).

In the case of a massive test particle, the geodesic equation (63) can be reduced to [33]

$$\mathcal{E} = \frac{1}{2}\left(\frac{dr}{d\tau}\right)^2 + V_{\text{eff}}(r), \tag{75}$$

where the effective "radial potential" is given by

$$V_{\text{eff}}(r) \equiv -\frac{M}{r} + \frac{\ell^2}{2r^2} - \frac{M\ell^2}{r^3} \qquad \text{(particle)}. \tag{76}$$

The "energy" term is given by

$$\mathcal{E} \equiv \frac{e^2 - 1}{2}, \tag{77}$$

where e and ℓ are the conserved quantities associated with translations of t ("energy") and ϕ ("angular momentum") defined in eq. (72).

The practical significance of eq. (75) is that it has the form of the total energy equation for one-dimensional motion in Newtonian mechanics, where the parameter $\mathcal{E}$ plays the role of the total energy, $\frac{1}{2}\left(\frac{dr}{d\tau}\right)^2$ that of the kinetic energy, and $V_{\text{eff}}(r)$ functions as an effective radial potential. Therefore, the qualitative aspects of the radial motion can be analyzed entirely by inspecting the diagram of $V_{\text{eff}}(r)$ at specific values of e and ℓ. In fact, in the non-relativistic limit and dropping the relativistic correction term $\frac{M\ell^2}{r^3}$ from (76), eq. (75) reduces to the exact expression of the Newtonian energy in terms of kinetic and gravitational potential energy.

Since the electronic quasi-particles in graphene are formally equivalent at the semi-classical level to photons, we are interested specifically in the propagation of light rays. In that case, the effective radial equation that derives from eqs. (66) and (67) becomes [33]

$$\frac{1}{b^2} = \frac{1}{\ell^2}\left(\frac{dr}{d\lambda}\right)^2 + W_{\text{eff}}(r), \tag{78}$$

where the effective potential now reads

$$W_{\text{eff}}(r) \equiv \frac{1}{r^2} - \frac{2M}{r^3} \qquad \text{(light)}, \tag{79}$$

and $b^2 \equiv \ell^2/e^2$ is the squared impact parameter of the light's trajectory. The study of effects such as gravitational lensing or the gravitational Doppler shift are most straightforwardly analyzed in the framework of this effective potential.

Trapping of light by a strong gravitational field At the qualitative level, this question is addressed by a simple analysis of the effective potential (78) for different values of the impact parameter. $W_{\text{eff}}(r)$ is plotted in Supplementary Figure **19**(a) and its key feature is the existence of a radial potential barrier. The effective potential has a maximum at $R_{\text{max}} = 3M$,

$$W(R_{\text{max}}) = \frac{1}{27M^2} \equiv W_{\text{max}}, \tag{80}$$

is attractive for $r < R_{\text{sc}}$, and singularly diverges as $\sim -1/r^3$. Remember that this potential arises from the *outer* Schwarzschild metric (68). Consequently, the plot is meaningful only in the region

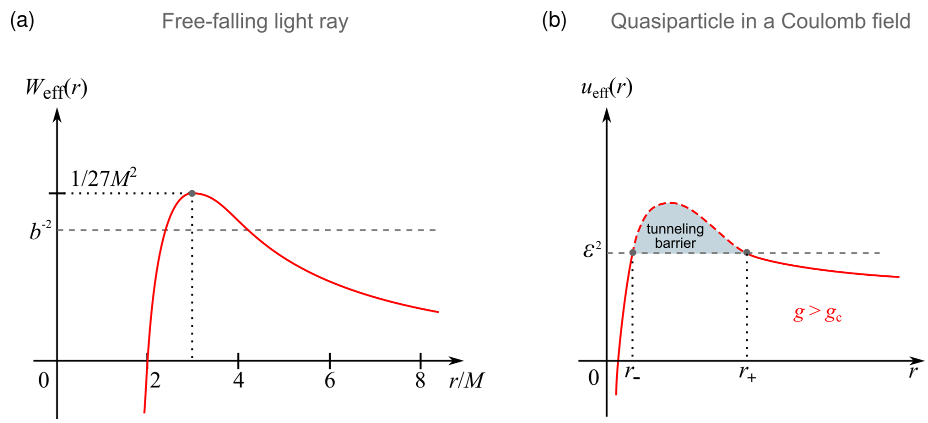

Supplementary Figure 19: (a) The (outer) effective geodesic potential $W_{eff}(r)$ defined in eq. (79) for a light ray free-falling under the influence of a spherically symmetric mass distribution. If the light ray is launched inside the region $r < 3M$ with impact parameter as depicted by the horizontal dashed line, it will be trapped by the radial potential barrier. (b) The effective semi-classical potential defined in eq. (55) for a Dirac quasi-particle in graphene in the presence of a Coulomb field.

$r > R$, where R is the extension of the mass; the effective potential in the region $0 < r < R$ must be replaced by whatever follows from the inner metric of that case. In particular, the region $r < R_{\text{sc}} = 2M$ is only meaningful in the case of a black hole because only then does R_{sc} lie outside the mass distribution ($R < R_{\text{sc}}$). In a black hole scenario, the centrifugal barrier $1/r^2$ is unable to counter the strongly attractive $1/r^3$ singularity, which is the reason no light ray escapes the gravitational pull once inside the Schwarzschild radius.

In a non-black-hole scenario where $R > R_{\text{sc}}$, the important difference is that there is no singular collapse towards $r \to 0$ because the $1/r^3$ singularity can be regularized in the inner space-time regions. Yet, if $R < R_{\text{max}}$, Supplementary Figure (**19**) shows that light rays with impact parameter satisfying $b^2 > 27M^2$ can still be trapped. The case of particular interest to us is when the inner region consists of a distribution of discrete masses in otherwise empty space (such as in a cluster of stars), in which case light rays can propagate in the inner region without necessarily being absorbed by reaching one of the masses. The different kinds of light ray trajectories in this case are hence:

1. Light aimed from $r = \infty$ with $b^{-2} < W_{\text{max}}$: the trajectory has a minimum distance of approach but continues to infinity in an hyperbolic orbit. This is the effect of light bending.

2. Light aimed from $r = \infty$ with $b^{-2} > W_{\text{max}}$: in the case of a black hole, the light ray approaches the mass distribution and never escapes. In a non-black hole scenario it is another case of light bending.

3. Light sent from $r = R_{\text{max}}$ with precisely $b^{-2} = W_{\text{max}}$: the light ray will trace a circular orbit at $r = R_{\text{max}}$. This orbit is unstable since it corresponds to a maximum of $W_{eff}(r)$.

4. Light sent from $r < R_{\text{max}}$ with $b^{-2} < W_{\text{max}}$: the light ray will be trapped inside the region $r < R_{\text{max}}$. In the case of a black hole, it inexorably collapses onto the star. In the case of a mass distribution with $R_{\text{sc}} < R < R_{\text{max}}$, the light ray might wander in the inner region $r < R$, depending on the specific details of the mass distribution within. In any case, the light ray is trapped.

13.3 Light vs the Coulomb attraction of massless Dirac particles in graphene

Figure **19**(b) represents the effective semi-classical potential (55) derived earlier for a Dirac quasi-particle in graphene near an unscreened Coulomb charge, and on the basis of which we established the existence of supercritical states as those that are trapped by the potential barrier. It is clear that there is no qualitative difference between the two potentials drawn in Supplementary Figures **19**(a) and **19**(b). Therefore, the semi-classical orbit of a hole in graphene near a negative Coulomb charge is qualitatively analogous to that of a light ray in the presence of a strong space-time curvature brought about by a dense mass distribution. Note that, rigorously, the behavior can be said to be "the same" at the qualitative level only: In the frustrated collapse regime, there are classical orbits that can be trapped in a finite region of space in both cases without them ever "collapsing" in any way.

The reason this analogy is adequate in the case of our experiments with discrete, subcritical molecular charges is that the physics pictured in Supplementary Figure **19** applies in the *far-field* of the charge distribution defined in the main text; the gravitational counterpart is a discrete cluster of massive stars. The fact that each charge is itself subcritical implies that the orbits of the charge carriers shown in Supplementary Figure **18**(b) spiraling towards collapse onto one charge are regularized in the *near-field* and become endlessly meandering paths confined within the charge distribution, as sketched in Fig. 5 of the main text. Formally, this is due to the fact that, when extended to include the details of the near-field, the effective potential depicted in Supplementary Figure **19**(b) will have the $1/r^2$ singularity cut-off at $r \sim d$ (cf. Supplementary Note 12 above). Analogously, if the star cluster is massive enough to have a Schwarzschild radius spanning a number of individual stars, light will be trapped within. In the case of the Coulomb charges, if one clumps them onto one single supercritical charge, those trapped orbits become collapsing trajectories that fall into the supercritical charge. In the case of light, clumping all the individual stars onto each other yields a black-hole, in which case the trajectories of light are also inevitably going to fall in.

But, at a more detailed level, there are differences. One is the fact that, in the case of gravity, the trapping is controlled by the competition between the terms $1/r^2$ and $-1/r^3$ in the (far-field) effective potential (79), whereas in the Coulomb case it is a competition between $1/r$ and $-1/r^2$, as per eq. (55). The different powers in the effective potential make the classical trajectories different at the fine quantitative level (for example, the equations of motion for the "particles"

described by these effective potentials will be different since the effective forces are different). Still, they will have the same *qualitative* nature: trapped orbits as a result of a singularly attractive term in the effective potential that overcomes the centrifugal barrier.

The other difference lies in the fact that, in the Coulomb case, the tunneling barrier persists irrespective of the energy of the particle. This happens because, as discussed in section 13.1, the term proportional to $1/r$ in the effective potential (55) depends explicitly on the energy in such a way that, no matter how low or high an energy is considered, the effective potential always looks as in Supplementary Figure **19**(b) and one cannot get rid of this barrier in the supercritical regime. The net result of this semi-classical feature is that, if an orbit starts in the inner region, it cannot escape to infinity in the supercritical Coulomb case (but it can always tunnel quantum-mechanically, which is the origin of the finite linewidth of the supercritical resonances). In contrast, the effective geodesic potential for the light/gravity case (eq. 78) depends only on the mass. This means that you can tune the impact parameter (or energy) independently and will have some orbits that can escape the gravitational pull. Therefore, the analogy strictly applies for the light rays with impact parameter corresponding to the trapped regime.

Supplementary References

[1] H. S. Jung et al., J. Vis. Exp. 101, e52711 (2015).

[2] G. V. Nazin, X. H. Qiu, and W. Ho, Phys. Rev. Lett. 95, 166103 (2005).

[3] N. A. Pradhan, N. Liu, C. Silien, and W. Ho, Phys. Rev. Lett. 94, 76801 (2005).

[4] F. Marczinowski, J. Wiebe, F. Meier, K. Hashimoto, and R. Wiesendanger, Phys. Rev. B 77, 115318 (2008).

[5] P. R. Wallace, Phys. Rev. 71, 622 (1947).

[6] A. H. Castro Neto, F. Guinea, N. M. R. Peres, K. S. Novoselov, and A. K. Geim, Rev. Mod. Phys. 81, 109 (2009).

[7] V. M. Pereira, J. Nilsson, and A. H. Castro Neto, Phys. Rev. Lett. 99, 166802 (2007).

[8] A. V. Shytov, M. I. Katsnelson, and L. S. Levitov, Phys. Rev. Lett. 99, 236801 (2007).

[9] A. V. Shytov, M. I. Katsnelson, and L. S. Levitov, Phys. Rev. Lett. 99, 246802 (2007).

[10] D. S. Novikov, Appl. Phys. Lett. 91, 102102 (2007).

[11] V. N. Kotov, B. Uchoa, V. M. Pereira, F. Guinea, and A. H. Castro Neto, Rev. Mod. Phys. 84, 1067 (2012).

[12] R. Haydock, in Solid State Phys. (Academic Press, New York, 1980), pp. 216–294.

[13] D. G. Pettifor and D. L. Weaire, The Recursion Method and Its Applications (Springer Berlin, 1987).

[14] E. Anderson, Z. Bai, C. Bischof, S. Blackford, J. Demmel, J. Dongarra, J. Du Croz, A. Greenbaum, S. Hammarling, A. McKenney, and D. Sorensen, LAPACK Users' Guide, Third (Society for Industrial and Applied Mathematics, Philadelphia, PA, 1999).

[15] Y. Zhang, V. W. Brar, F. Wang, C. Girit, Y. Yayon, M. Panlasigui, A. Zettl, and M. F. Crommie, Nat. Phys. 4, 627 (2008).

[16] V. W. Brar, S. Wickenburg, M. Panlasigui, C.-H. Park, T. O. Wehling, Y. Zhang, R. Decker, C. Girit, A. V. Balatsky, S. G. Louie, A. Zettl, and M. F. Crommie, Phys. Rev. Lett. 104, 36805 (2010).

[17] B. Wunsch, T. Stauber, F. Sols, and F. Guinea, New J. Phys. 8, 318 (2006).

[18] T. Ando, J. Phys. Soc. Jpn. 75, 74716 (2006).

[19] E. H. Hwang and S. Das Sarma, Phys. Rev. B 75, 205418 (2007).

[20] M. I. Katsnelson, Phys. Rev. B - Condens. Matter Mater. Phys. 74, 201401 (2006).

[21] I. S. Terekhov, A. I. Milstein, V. N. Kotov, and O. P. Sushkov, Phys. Rev. Lett. 100, 76803 (2008).

[22] A. F. Young, C. R. Dean, I. Meric, S. Sorgenfrei, H. Ren, K. Watanabe, T. Taniguchi, J. Hone, K. L. Shepard, and P. Kim, Phys. Rev. B 85, 235458 (2012).

[23] K. K. Kim, A. Hsu, X. Jia, S. M. Kim, Y. Shi, M. Dresselhaus, T. Palacios, and J. Kong, ACS Nano 6, 8583 (2012).

[24] Y. Wang, D. Wong, A. V Shytov, V. W. Brar, S. Choi, Q. Wu, H.-Z. Tsai, W. Regan, A. Zettl, R. K. Kawakami, S. G. Louie, L. S. Levitov, and M. F. Crommie, Science 340, 734 (2013).

[25] S. Wickenburg, J. Lu, J. Lischner, H.-Z. Tsai, A. A. Omrani, A. Riss, C. Karrasch, A. Bradley, H. S. Jung, R. Khajeh, D. Wong, K. Watanabe, T. Taniguchi, A. Zettl, A. H. C. Neto, S. G. Louie, and M. F. Crommie, Nat. Commun. 7, 13553 (2016).

[26] O. O. Sobol, E. V. Gorbar, and V. P. Gusynin, Phys. Rev. B 88, 205116 (2013).

[27] D. Klöpfer, A. De Martino, D. U. Matrasulov, R. Egger, Eur. Phys. J. B. 87, 187 (2014).

[28] V. M. Pereira, V. N. Kotov, and A. H. Castro Neto, Phys. Rev. B 78, 85101 (2008).

[29] V. N. Kotov, V. M. Pereira, and B. Uchoa, Phys. Rev. B 78, 75433 (2008).

[30] Y. B. Zeldovich and V. S. Popov, Sov. Phys. Uspekhi 14, 673 (1972).

[31] W. Greiner, B. Müller, and J. Rafelski, Quantum Electrodynamics of Strong Fields, 2nd ed. (Springer Berlin, 1985).

[32] A. H. Castro Neto, F. Guinea, N. M. R. Peres, K. S. Novoselov, A. K. Geim, The electronic properties of graphene. Rev. Mod. Phys. 81, 109 (2009).

[33] J. B. Hartle, Gravity: An introduction to Einstein's General Relativity (Addison-Wesley, San Francisco, 2003).
